# Yad fimbriae are triggered by host cues and enhance extraintestinal pathogenic *Escherichia coli* tissue colonisation during bloodstream infection

**DOI:** 10.1371/journal.ppat.1014299

**Published:** 2026-06-01

**Authors:** Chloe Ellison, Curtis Cottam, Zheng Jie Lian, Tugce Onur, Burhan A. Choudhry, Yvette Yu Ting Ong, Minh-Duy Phan, Mark A. Schembri, James P. R. Connolly

**Affiliations:** 1 Newcastle University Biosciences Institute, Newcastle University, Newcastle-upon-Tyne, United Kingdom; 2 Institute for Molecular Bioscience, The University of Queensland, Brisbane, ‌‌Queensland, Australia; 3 Australian Infectious Diseases Research Centre, The University of Queensland, Brisbane, ‌‌Queensland, Australia; 4 School of Chemistry and Molecular Biosciences, The University of Queensland, Brisbane,‌‌ Queensland, Australia; Deutsches Elektronen-Synchrotron, GERMANY

## Abstract

Bacterial pathogens that infect host sites beyond their native ecological niche must be equipped to cope with unique challenges across distinct environments. This often manifests in the upregulation of virulence factors specifically in response to host cues, which enhance pathogen fitness. Extraintestinal pathogenic *Escherichia coli* (ExPEC) typically colonise the host-gut asymptomatically but can disseminate to infectious sites such as the bladder, kidneys and bloodstream. The molecular basis of urinary tract colonisation by ExPEC is well established, with adhesion via chaperone-usher fimbriae being a critical determinant. However, mechanisms that promote bloodstream infection are poorly understood. Here, we show that several ExPEC fimbriae are upregulated rapidly in response to human serum, mimicking exposure to the bloodstream environment. Yad fimbriae displayed the most significant induction in response to this host cue in two distinct ExPEC isolates, and we show that the gene cluster is prevalent across the *E. coli* phylogeny, suggesting a common virulence mechanism. Expression of Yad fimbriae was found to be repressed at the transcriptional level by the histone-like nucleoid structuring protein (H-NS). Furthermore, a prolonged elevation in Yad transcription was sustained throughout many generations of growth in serum, suggesting that cue(s) in the bloodstream counteract H-NS repression, triggering cell-surface expression of Yad fimbriae. Finally, Yad transcription was significantly upregulated within systemic tissue in a murine model of bacteremia and we show that deletion of the *yad* genes significantly attenuated ExPEC colonisation during infection. These data reveal Yad fimbriae as an important ExPEC virulence factor and support the concept of cellular adhesion as a crucial element of bacterial bloodstream pathogenesis.

## Author summary

Extraintestinal pathogenic *Escherichia coli* (ExPEC) colonise the human gut without causing disease but can disseminate to the urinary tract and bloodstream. Understanding the mechanisms that enable ExPEC survival in the bloodstream is key to devising novel strategies that block this process. Here, we have discovered that exposure to human serum acts as a cue to trigger expression of Yad fimbriae, a cell-surface appendage that is widely encoded across the *E. coli* species and promotes adhesion to host tissue. We show that expression of Yad fimbriae is blocked at the transcriptional level in the absence of host cues and that deletion of the genes encoding Yad reduces the severity of bloodstream infection in mice. Our data identify Yad fimbriae as an important ExPEC virulence factor, supporting the concept of tissue adhesion being a key mechanism driving bloodstream pathogenesis.

## Introduction

Extraintestinal pathogenic *E. coli* (ExPEC) is a major human pathogen capable of infecting multiple, distinct host-niche [1–3]. ExPEC colonises the gut asymptomatically but can infect the urinary tract, ascend to the kidneys and enter the bloodstream where it disseminates systemically [4–7]. ExPEC can also translocate directly from the gut to the bloodstream [3]. As such, ExPEC, a broad pathotype that encompasses uropathogenic *E. coli* (UPEC), is the primary causative agent of urinary tract infections (UTI) and bloodstream infections (BSI), responsible for ~75% and ~25% of all cases, respectively [1,2]. ExPEC isolates are also frequently resistant to multiple antibiotics and were responsible for ~23% of the 1.27 million deaths globally attributed to antimicrobial resistance in 2019 [8,9]. This has led to ExPEC that are resistant to third-generation cephalosporins and carbapenems being ranked as a critical group of bacterial priority pathogens by the World Health Organisation (https://www.who.int/publications/i/item/9789240093461).

ExPEC employ an array of virulence and fitness enhancing factors to cause infection, with host cell adhesion being one of the key mechanisms driving this process [1,10,11]. ExPEC adhesion is primarily mediated by chaperone-usher pathway fimbriae [12,13]. These polymeric surface structures are assembled via a combined periplasmic chaperone and cognate outer membrane usher apparatus, which mediate the assembly of the > 1000 subunits that constitute individual fimbriae [14]. The fimbriae are capped with a receptor binding tip adhesin, that contains an N-terminal lectin domain mediating adhesion to specific ligands present on the surface of host cells. While several ExPEC chaperone-usher systems have been characterised, the most well-studied example is Type 1 fimbriae, which plays a key role in adhesion of ExPEC to the bladder via a specific interaction between the tip-associated FimH adhesin and α-D-mannosylated glycoproteins decorating the surface of the bladder epithelium [15–19]. However, ExPEC strains typically encode multiple other chaperone-usher fimbriae loci located across the core and accessory genome, which vary in combination between different strains [20,21]. The precise molecular mechanisms that govern the regulation, expression, receptor interactions and role in infection of some of these other fimbriae are not completely understood [22–24].

While extensive research exists describing the details underlying ExPEC interaction with the urinary tract and the gut, much less is known about the mechanisms that promote BSI [2]. The bloodstream represents an incredibly complicated infectious environment, where ExPEC must navigate dramatic shifts in metabolite availability, interaction with physiologically diverse organ structures and cell types, and avoiding killing by cellular and soluble innate immune factors. We previously discovered Type 1 and F1C fimbriae were co-ordinately upregulated in response to human serum, which mimics signals encountered upon entry into the bloodstream [25]. Accordingly, both factors were upregulated in host tissue and contributed to the competitive fitness of ExPEC in a murine infection model. This suggests that host-cell adhesion is a key mechanism underlying the ability of ExPEC to establish a BSI. Here, we screened all chaperone-usher fimbriae from two distinct ExPEC isolates in response to human serum and discovered that Yad fimbriae showed the most significant induction. We further show that the *yad* fimbriae locus is widely encoded across the *E. coli* species and frequently found in ExPEC strains, is tightly regulated by a global repressor protein, and that deletion of the genes encoding Yad from two different ExPEC strains attenuated BSI in a mouse infection model. Together, these data support a key role for fimbriae-mediated adhesion as a critical mechanism to maximise ExPEC fitness during BSI, with Yad fimbriae emerging as an important mediator of this process.

## Results

### Exposure of ExPEC to human serum triggers transcription of multiple chaperone-usher fimbriae loci

**Fig 1:**
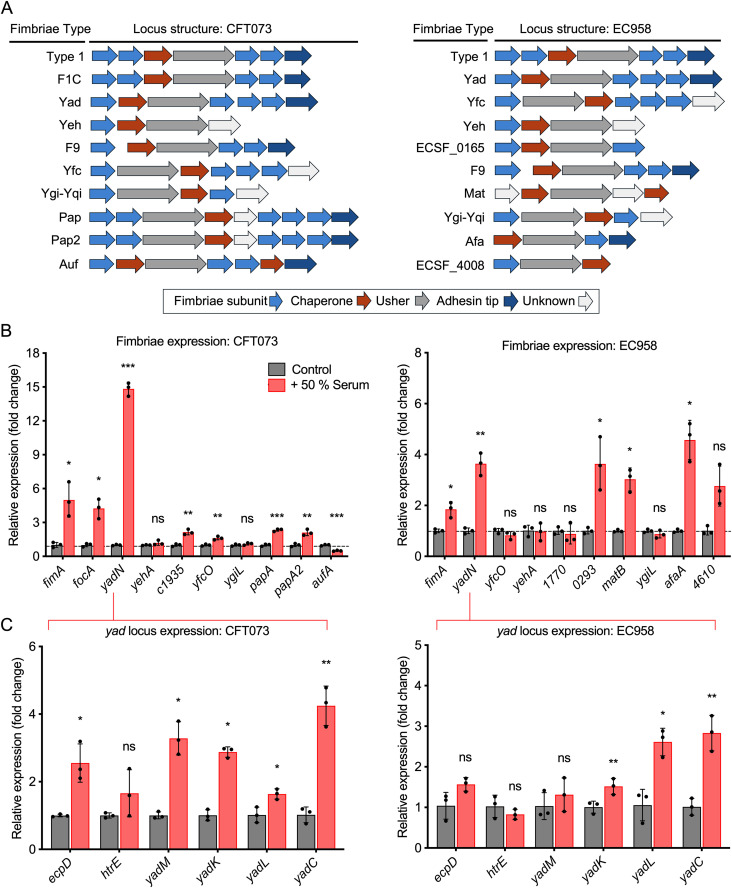
Human serum triggers transcription of multiple CU fimbriae loci. **(A)** Schematic illustration of all chaperone-usher loci in ExPEC strains CFT073 and EC958. The fimbriae type is named on the left of each panel and a key depicting the components of each system illustrated underneath. **(B)** qRT-PCR analysis of fimbriae subunit genes representing each system from CFT073 and EC958. Cells were cultured in MEM-HEPES and spiked with either 50% PBS as the baseline control (grey) or human serum (red) for 20 minutes prior to RNA extraction and cDNA conversion. **(C)** qRT-PCR analysis of individual *yad* locus genes from CFT073 and EC958 cultured in MEM-HEPES spiked with either 50% PBS or human serum as above. The bars depict the relative fold change above the control (grey bars) and the error bars represent the standard deviation (n = 3). *, **, *** or ns indicate *P* < 0.05, *P* < 0.01, *P <* 0.001 or not significant, respectively, as determined by a two-tailed t-test.

In our previous work, we discovered that two distinct fimbrial loci (Type 1 and F1C) were transcriptionally upregulated in response to human serum [25]. This led us to show that deletion of either system resulted in a fitness decrease during experimental BSI. We therefore hypothesised that other fimbriae may be similarly regulated and thus enhance systemic fitness of ExPEC during BSI. We began by examining the transcription of all chaperone-usher fimbriae systems in two well-characterised ExPEC strains – the urosepsis isolate CFT073 (ST73) and the UTI isolate EC958 (ST131) [20,26–28]. We performed qRT-PCR to assess the transcript level of the major fimbrial subunit gene as a representative of each chaperone-usher locus in these strains (Fig 1A). RNA was extracted from cells cultured in MEM-HEPES to mid-log phase (OD_600_ = ~0.5) that was spiked with 50% human serum or sterile PBS as a control for 20 minutes, mimicking the rapid responses that occur after transition into the bloodstream during an infection. Note that these ExPEC strains are resistant to the bactericidal effects of serum and no loss of cell viability was observed in response to human serum (S1 Fig). Strikingly, we found that several fimbriae loci in both strains (7/10 for CFT073; 5/10 for EC958) were upregulated in response to human serum, with some responses being strain-specific and only one system (Auf) displaying downregulation (Fig 1B). Importantly, the expected upregulation of Type 1 fimbriae, and the fimbriae loci showing no response to serum (Yeh and Ygi-Yqi), were conserved between the strains suggesting that the regulatory response to host cues is consistent and specific. Our attention was drawn to *yadN*, encoding the major fimbrial subunit of the Yad system, which showed the strongest response of all chaperone-usher systems to human serum (15-fold/*P* = 0.0006 in CFT073; 4-fold/*P* = 0.0064 in EC958). The *yad* locus encodes the major subunit YadN as well as the EcpD chaperone, the HtrE usher, the YadMLK minor subunits and the YadC tip adhesin [13,29,30]. Further qRT-PCR analysis of each corresponding gene showed the majority of the *yad* locus was upregulated in response to serum for both strains. Interestingly, we noticed that certain genes displayed a more significant increase than others, suggesting differential regulation within the *yad* locus itself (Fig 1C). Indeed, differential processing and expression of genes within fimbrial loci has been observed previously by us and others [31–33]. These data suggest a generalised response to host-derived cues found in the bloodstream and indicates that Yad fimbriae may represent an important virulence factor providing a fitness advantage during BSI.

### Carriage of the Yad fimbriae locus is prevalent across the *E. coli* species

**Fig 2:**
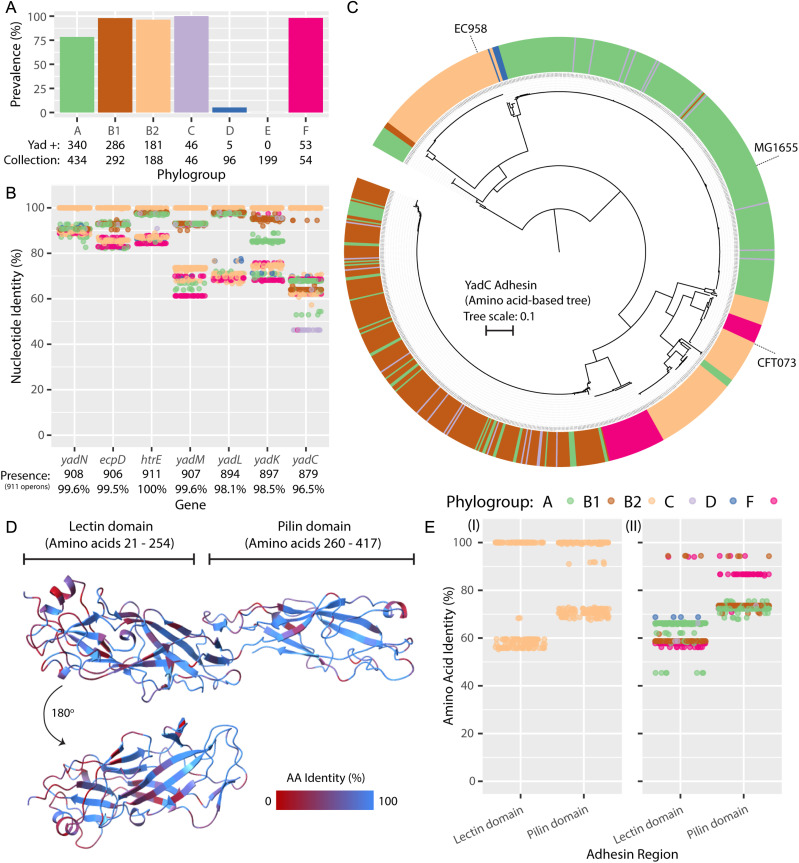
Prevalence of Yad fimbriae across the *E. coli* species. **(A)** Yad operon prevalence amongst 1310 completely sequenced *E. coli* genomes by phylogroup. Strains containing the *htrE* usher at an 80% alignment length and identity threshold in a tBLASTn search (HtrE^EC958^ query) were considered Yad positive. **(B)** Nucleotide conservation and prevalence of each gene in the 911 *yad* operons. Amino acid sequences from the Yad operon of EC958 were used in a tBLASTn search against the 911 *yad* operons with an 80% alignment length threshold. **(C)** Maximum likelihood phylogeny of the *yadC* adhesin. Complete YadC adhesin coding sequences from the 911 *yad* operons were translated, aligned, and subsequently utilised to generate a maximum likelihood phylogeny (100 bootstraps) using CLC Main Workbench 23. The phylogeny was visualised as a midpoint rooted tree using the interactive Tree of Life. EC958, CFT073, and MG1655 references are noted. The tree scale represents amino acid substitutions per site. **(D)** Predicted structure of YadC^EC958^ with the conservation of each residue from the 911 *yad* operons. The predicted structure of YadC^EC958^ was generated using ColabFold. Amino acid conservation for each residue corresponding to YadC^EC958^ was calculated based on similarity using the BLOSUM62 matrix and mapped onto YadC^EC958^ using ChimeraX (v1.10.1), with alignment gaps excluded from visualisation. **(E)** Amino acid identities of the lectin and pilin domain of YadC separated into (I) phylogroup B2 and (II) other phylogroups. The lectin and pilin domains of YadC^EC958^ were used in a BLASTp search against predicted YadC primary sequences from the 911 Yad operons at an 80% length threshold.

The prevalence of *yad* fimbriae across the *E. coli* species was next assessed by interrogating a dataset of 1310 completely sequenced *E. coli* genomes from NCBI RefSeq (20/01/2021). Strains were classified as Yad positive if they encoded the conserved usher protein HtrE^EC958^. In total, 70% (911/1310) of the strains were Yad positive, with high prevalence noted in phylogroups C (100%; 46/46), F (98.1%; 53/54), B1 (97.9%; 286/292), B2 (96.3%; 181/188), and A (78.3%; 340/434) (Fig 2A). Only a small proportion of phylogroup D was Yad positive (5.2%; 5/96), and no strains from phylogroup E contained Yad fimbriae genes (Fig 2A). The MLST-based distribution of Yad positive strains is presented in the supplementary information (S1 Table). To gain a wider perspective of Yad fimbriae prevalence across *E. coli*, we further examined Yad presence in isolates separated by the following source niches: companion animals, environment, food, humans, livestock, poultry, and wild animals. Across all niches, Yad fimbriae were highly prevalent, with >75% of strains carrying the conserved *htrE*-usher encoding gene (S2 Fig).

Next, the conservation of each gene within the *yad* operon of the 911 Yad-positive strains was assessed using the EC958 *yad* operon as a tBLASTn query. Our analyses revealed that most *yad* operons contained a homolog corresponding to the major subunit YadN (99.6%), the chaperone EcpD (99.5%), the minor subunits YadMLK (98.5-99.6%), and the adhesin YadC (96.5%) (Fig 2B). However, genes encoding different fimbriae components had different levels of nucleotide sequence variation. The major subunit, chaperone, and usher components had low sequence variation, with nucleotide conservation ranging from 80-100% (Fig 2B). Conversely, the minor subunits and adhesin had high nucleotide sequence variation, ranging from 60-100% and 45–100%, respectively (Fig 2B). When analysed by phylogroup, we also observed two distinct Yad fimbriae subtypes present in phylogroup B2 (Fig 2B; S3 Fig).

The sequence variation of the YadC adhesin was explored further. A phylogenetic analysis of complete YadC sequences (from the 911 Yad-positive collection) revealed clustering largely driven by phylogroup, with evidence of recombination between phylogroups (Fig 2C). YadC sequences from phylogroup B2 were split into two distinct clusters representative of the reference UPEC strains EC958 and CFT073 (Fig 2C & 2B).

The YadC adhesin possesses a classical two-domain fimbrial adhesin structure comprising an N-terminal ligand binding lectin domain (involved in receptor binding) and a C-terminal pilin domain (involved in organelle attachment). Thus, we also investigated amino acid conservation across these two domains. After mapping the conservation of each residue to the YadC^EC958^ adhesin, we observed reduced conservation across residues in the lectin domain (Fig 2D). These differences were quantified using the lectin and pilin domains of YadC^EC958^ in separate BLASTp searches against the 911 *yad* operons. In phylogroup B2 (Fig 2E; panel I), the two distinct YadC homologs are again evident, where one has almost exact identity to YadC^EC958^, and the second has ~ 60% and ~70% identity across the lectin and pilin domains, respectively. For the other phylogroups, the lectin domain consistently has lower amino acid identity compared to the pilin domain (Fig 2E; panel II). Taken together, our analyses reveals that the *yad* operon is broadly distributed across *E. coli* isolated from different ecological niches, across the phylogroups A, B1, B2, C, and F, with the major subunit, chaperone, and usher showing strong sequence conservation. In contrast, the minor subunits and the adhesin exhibit substantial sequence variation, particularly within the lectin domain of the YadC adhesin, possibly reflecting differences in binding specificity.

D-xylose is known to be a ligand for YadC [30, 34]. To investigate if variation in the YadC lectin domain impacts its receptor binding affinity, we docked D-xylose to the YadC lectin domain of K-12 MG1655 (YadC-LD^MG1655^), EC958 (YadC-LD^EC958^), and CFT073 (YadC-LD^CFT073^). As the binding site is undefined, we used FPocket to identify a conserved pocket across all three proteins near the N-terminus containing key residues tryptophan, alanine, and valine. Docking of D-xylose into the pocket yielded differences in binding affinity for YadC-LD^MG1655^ (-3.248kcal/mol), YadC-LD^EC958^ (-4.358kcal/mol) and YadC-LD^CFT073^ (-3.853kcal/mol) (S4 Fig). YadC-LD^EC958^ formed additional contacts with asparagine and glycine (S4B Fig), consistent with its predicted stronger binding compared to YadC-LD^MG1655^ (S4A Fig), whereas YadC-LD^CFT073^ variation in the binding pocket (lysine instead of aspartic acid and added serine) reduced predicted affinity relative to YadC-LD^EC958^ but increased affinity versus YadC-LD^MG1655^ (S4C Fig). Overall, these in silico predictions suggest variation in the YadC lectin domain may impact receptor binding affinity.

### The *yad* locus is repressed by the nucleoid-associated H-NS protein in ExPEC

**Fig 3:**
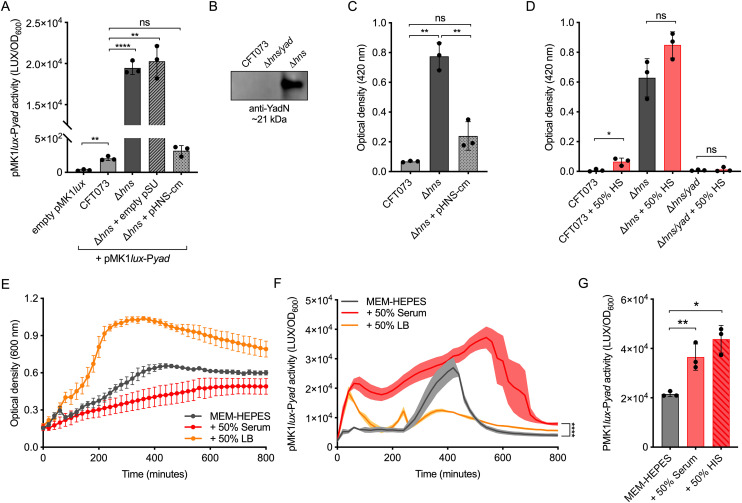
H-NS represses the Yad expression in ExPEC in the absence of host cues. **(A)** Transcriptional reporter assay of CFT073, Δ*hns,* Δ*hns* + empty pSU2718 (pSU) and Δ*hns* + pHNS-cm transformed with the pMK1*lux-*P*yad* reporter plasmid. The empty vector control corresponds to CFT073 transformed with promoterless pMK1*lux*. Data are depicted as relative luminescence units (LUX) divided by the optical density (600 nm) of cultures grown in MEM-HEPES and sampled at mid log phase. The error bars represent the standard deviation (n = 3).**, **** or ns indicates *P <* 0.01, *P* < 0.00001 or not significant, respectively, as determined by a two-tailed t-test. **(B)** Western blot analysis of YadN expression from cultures of CFT073, Δ*hns* or Δ*hns/yad* cells grown in MEM-HEPES. The result is representative of three biological replicates. **(C)** Whole cell ELISA detection of YadN cell-surface expression in CFT073, Δ*hns* or Δ*hns* + pHNS-cm cultured in MEM-HEPES. **(D)** Whole cell ELISA detection of YadN cell-surface expression in CFT073, Δ*hns* or Δ*hns/yad* cultured in MEM-HEPES (grey bars) or MEM-HEPES supplemented with 50% human serum (red bars). For ELISAs, the error bars represent the standard deviation (n = 3). *, ** or ns indicate *P* < 0.05, *P* < 0.01 or not significant, respectively, as determined by a two-tailed t-test. **(E)** Growth curve analysis of Δ*hns* transformed with pMK1*lux-*P*yad* cultured in MEM-HEPES alone (grey) and the same media supplemented with 50% human serum (red) or 50% LB media (yellow). The error bars represent the standard deviation (n = 3). **(F)** Temporal transcriptional reporter assay of Δ*hns* transformed with pMK1*lux-*P*yad* cultured as per the conditions in panel **D.** The data are depicted as relative luminescence units (LUX divided by optical density) and the error shading represents the standard deviation (n = 3). **** indicates *P* < 0.0001 of the grey versus red data as determined by a two-tailed ANOVA. **(G)** Transcriptional reporter assay of Δ*hns* transformed with the pMK1*lux-*P*yad* reporter plasmid depicting LUX/OD_600_ measurements at late log phase from cultures grown in MEM-HEPES with either 50% human serum or heat-inactivated serum (HIS). The error bars represent the standard deviation (n = 3).* or ** indicates *P <* 0.05 or *P* < 0.01, respectively, as determined by a two-tailed t-test.

To study the dynamics of *yad* regulation in ExPEC, we generated a transcriptional reporter plasmid containing the *yadN* promoter region fused to the LUX operon of *Photorhabdus luminescens* (pMK1*lux-*P*yad*). We noticed that while relative luminescence levels from this system were greater than the baseline noise detected from a promoter-less pMK1*lux* control (*P* = 0.0054), *yad* promoter expression was low in the wild type CFT073 background cultured in MEM-HEPES (Fig 3A). It has been previously reported that the *yad* locus is repressed by the histone-like nucleoid structuring protein (H-NS) in non-pathogenic *E. coli* K-12, which could explain this result [30,35]. Indeed, examination of published H-NS ChIP-seq data for CFT073 identified that H-NS does bind the *yad* region in this strain [36]. To test this, we generated a Δ*hns* mutant in CFT073 and transformed pMK1*lux-*P*yad* into this genetic background. Loss of H-NS resulted in *yad* promoter activity increasing by >2 orders of magnitude (*P* < 0.0001), confirming this regulatory mechanism in ExPEC (Fig 3A). We validated this result at the transcript level by qRT-PCR, confirming that transcription of all genes within the *yad* operon (*yadN-ecpD-htrE-yadMLKC*) was significantly increased in the Δ*hns* background for both CFT073 and EC958, again observing different patterns of *yad* transcription in each strain (S5 Fig). Furthermore, we successfully complemented this altered *yad* transcription by constitutively expressing H-NS *in trans* from plasmid pSU2718 (pHNS-cm) in the reporter strain [37], thus restoring baseline levels of *yad* promoter activation (Fig 3A). Importantly, empty pSU2718 had no unexpected effect on reporter activity, thus validating the complementation strategy. To determine Yad expression at the translational and cellular levels, we first performed western blot analysis using antibodies against the major subunit YadN. This confirmed an absence of detectable YadN expression in wild type CFT073 but identified a strong ~21 kDa band in normalised Δ*hns* lysates (Fig 3B). As a control, we generated a Δ*hns*/*yad* double mutant, which resulted in loss of YadN detection. Next, to examine whether expression of YadN in the Δ*hns* background corresponds to fimbrial production on the cell surface, we used a whole-cell ELISA. As expected, based on our transcriptional data, detection of YadN surface expression was low on wild type CFT073 cells but >10-fold higher in Δ*hns* (*P =* 0.0053). YadN expression was successfully reversed by complementation using pHNS-cm to levels not significantly (*P* = 0.0905) higher than the wild type (Fig 3C). Furthermore, YadN surface expression was significantly increased in CFT073 cultured in MEM-HEPES with 50% human serum (*P* = 0.0432) but still far below that observed in Δ*hns* (Fig 3D)*.* Exposing Δ*hns* to 50% human serum in the medium also resulted in increased cell-surface expression of YadN, trending towards statistical significance (*P* = 0.0785). Importantly, YadN expression in the Δ*hns/yad* control strain did not increase above background levels during serum exposure (*P =* 0.5702). Similar results were observed in EC958, suggesting that H-NS regulation of Yad fimbriae is a common mechanism in ExPEC (S6 Fig).

Having validated that H-NS was a repressor of the *yad* locus in ExPEC, we next investigated the dynamics of *yad* promoter activity during growth in media containing 50% human serum using CFT073 Δ*hns* transformed with the reporter plasmid pMK1*lux-*P*yad*. Growth of this strain in MEM-HEPES with 50% human serum was slower than that observed in MEM-HEPES alone (Fig 3E). However, *yad* promoter activity was significantly higher in response to media containing human serum (*P* < 0.0001). Enhanced *yad* promoter activity was observed throughout all phases of the growth period, suggesting that the rapid transcriptional response of the Yad system to cues derived from human serum (as observed in Fig 1B) was sustained over several generations in the continued presence of serum (Fig 3F)*.* As a control, we performed the same experiment using MEM-HEPES supplemented with 50% LB. Growth was markedly faster under this condition (Fig 3D), and while an initial spike in *yad* promoter activity was observed, this response rapidly declined to levels lower than those measured in MEM-HEPES alone (Fig 3F). To address whether serum-induced activation of the *yad* promoter was complement-dependent, we also measured pMK1*lux-*P*yad* expression in response to heat-inactivated serum. This experiment identified no difference in the observed phenotype, indicating that active complement was not responsible for increasing *yad* expression (Fig 3G). Collectively, these results support the hypothesis that enhanced Yad expression was a condition-specific response of ExPEC after exposure to human serum and suggests that cue(s) encountered within the host during BSI can overcome H-NS mediated repression of the *yad* fimbriae locus.

### Yad fimbriae are upregulated in tissue and required for maximal fitness during BSI

**Fig 4:**
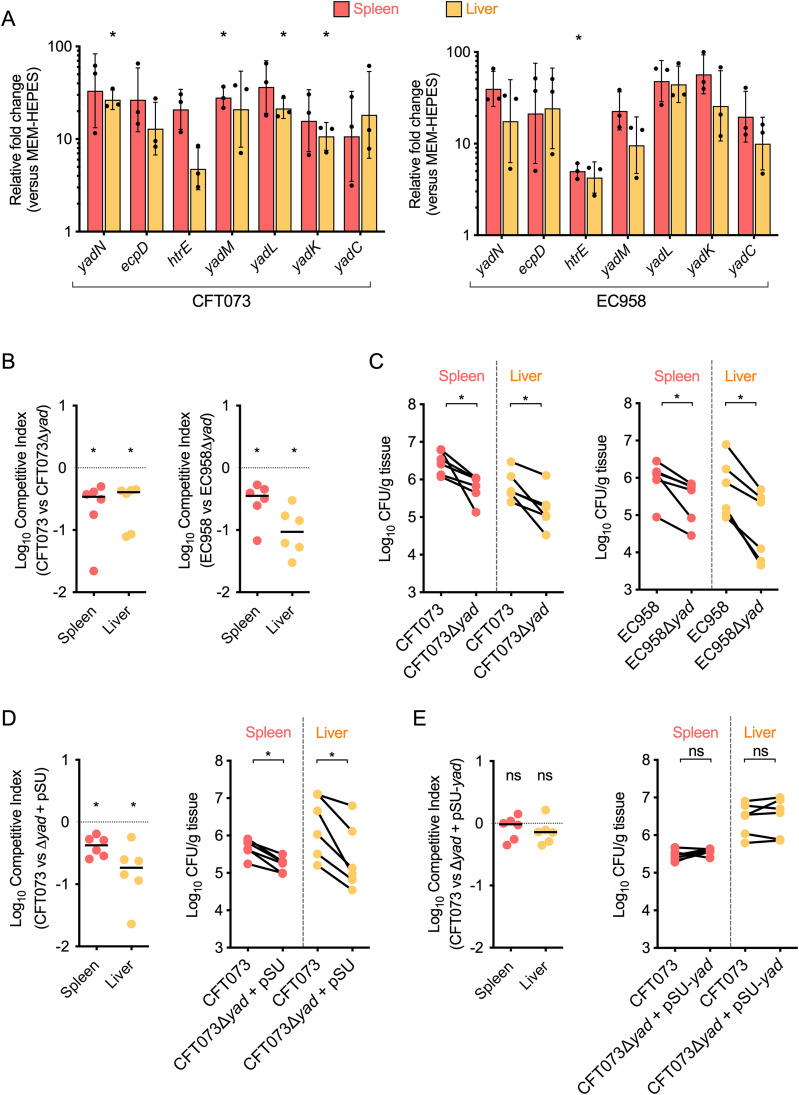
Yad fimbriae promote ExPEC fitness during bloodstream infection. **(A)** qRT-PCR analysis of relative *yad* locus gene transcript levels in RNA purified from splenic (red) or hepatic (yellow) tissue of female BALB/c mice intravenously infected with ExPEC strains CFT073 (left panel) or EC958 (right panel). The bars depict the relative fold change above cells cultured in MEM-HEPES (baseline of 1 on the x-axis) and the error bars represent the standard deviation (n = 3). * Indicates *P* ≤ 0.05, as determined by a repeated measures ANOVA with Dunnett’s post-test for multiple comparisons. **(B)** Competitive index of CFT073 (left panel) and EC958 (right panel) versus the corresponding *yad* locus deletion mutant (CFT073Δ*yad* and EC958Δ*yad* respectively) during murine bloodstream infection. **(C)** Paired CFU per organ within individual animals determined for each wild type and corresponding Δ*yad* mutant strain tested in the infections. **(D)** Competitive index (left panel) and paired CFU per organ of CFT073 versus CFT073Δ*yad* transformed with pSU2718 (pSU) as an empty vector control. **(E)** Competitive index (left panel) and paired CFU per organ of CFT073 versus CFT073Δ*yad* transformed with pSU-*yad*. For all *in vivo* competition experiments, female BALB/c mice (n = 6 per strain) were intravenously infected with a 1:1 mixture of wild type and mutant or complemented strains. After a 24-hour period the mice were sacrificed and organs removed for CFU determination of both strains. For calculation of the competitive index * Indicates *P* < 0.05 as determined by a Wilcoxon signed rank test. For paired CFU determination, * Indicates *P* < 0.05 as determined by a Wilcoxon matched-pairs signed rank test. ns indicates not significant for either test.

We hypothesised that due to Yad fimbriae being upregulated in response to serum, which mimics entry into the bloodstream, this adhesive organelle may provide an advantage for tissue colonisation during systemic BSI. To address this, we first purified mRNA from both splenic and hepatic tissue of female BALB/c mice intravenously infected with CFT073 or EC958 (n = 3 each). qRT-PCR analysis of *yad* gene levels from each strain compared to growth in MEM-HEPES revealed that the locus is consistently upregulated *in vivo* at both tissue sites. Relative expression levels were > 50-fold enhanced compared to *in vitro* growth for certain genes, with several genes displaying significant upregulation (Fig 4A). This suggests that H-NS mediated repression of the *yad* locus is relieved *in vivo,* promoting tissue colonisation after acute bloodstream entry*.* To test whether Yad fimbriae contribute to tissue colonisation, we next generated full deletions of the *yad* locus in both CFT073 and EC958, showing that these mutants did not exhibit a fitness defect during *in vitro* growth (S7 Fig). We next determined the relative competitive fitness of these mutants *in vivo,* by co-infecting mice with a 1:1 mixture of each mutant and their respective parental strain via direct injection into the tail vein. Mice were sacrificed after 24 hours of infection, and the colonisation burden was determined by CFU counts of homogenised organ lysates. Both CFT073Δ*yad* and EC958Δ*yad* mutants were significantly outcompeted by their respective parental wild type strains for colonisation of both the liver and spleen, with EC958Δ*yad* displaying the largest fitness defect in the liver of ~10-fold (Fig 4B). Importantly, the colonisation defect was consistent across all animals tested (Fig 4C; *P* = 0.0312).

To validate this fitness defect, we cloned the entire *yad* locus into pSU2718 (pSU-*yad*) and confirmed that this plasmid facilitates expression of functional Yad fimbriae by whole-cell ELISA (S8 Fig). We next co-infected mice with a 1:1 mixture of wild type CFT073 and Δ*yad* transformed with either pSU-*yad* or empty pSU2718 as a control. Note that subculturing strains carrying pSU-*yad* grown statically at 37 °C in LB over 3 days showed that the plasmid was > 90% stable without selection (S9 Fig), confirming its utility for *in vivo* complementation studies over a 24-hour infection period. The Δ*yad* mutant carrying empty pSU2718 resulted in a similar fitness defect observed for the mutant alone at both infection sites, suggesting that this backbone did not negatively affect fitness in a manner that could confound the results due to the lack of a plasmid matched wild type control (Fig 4D). However, Δ*yad* carrying pSU-*yad* successfully complemented this fitness defect, as indicated by an insignificant difference in CFUs recovered to that of wild type and loss of a competitive fitness defect (Fig 4E). Collectively, these data provide experimental evidence to support a role for Yad fimbriae as an important ExPEC virulence factor required for maximal fitness during BSI and suggests that cues derived from the bloodstream act as a molecular trigger to signal the upregulation of this system during human BSI*.*

## Discussion

Bacterial virulence factors are crucial determinants of a pathogens ability to cause disease. The utility of these factors often corresponds with the precise host niche colonised by the pathogen, and are triggered in response to the environment to provide a benefit. As the bloodstream represents an incidental infectious niche that is normally devoid of microbes, factors that promote BSI are traditionally thought to be focused on immune evasion (e.g., complement resistance mechanisms) or nutrient acquisition [2,7,38–40]. However, recent discoveries have highlighted fimbriae as being potentially important factors in driving BSI [4,25]. Here, we discovered that many chaperone-usher fimbriae in ExPEC are upregulated immediately after exposure to signals encountered in the bloodstream. Focusing on the Yad system, we show that these fimbriae are widely encoded by ExPEC strains, are tightly regulated under non-permissive conditions and enhance ExPECs ability to colonise host tissue during BSI. These findings support the notion that cellular adhesion is a crucial element of ExPEC BSI pathogenesis.

Type 1, P and F1C fimbriae also contribute to ExPEC BSI [4,25]. While defined receptors of these systems are known, including mannosylated uroplakins (Type 1 fimbriae), Galα1–4Gal-glycosphingolipids (P fimbriae) and galactosylceramides or globotriaosylceramides (F1C fimbriae), these interactions have been almost exclusively studied in the context of UTI and the precise tissue interactions of these fimbriae during BSI is unknown [16,41–43]. Yad fimbriae have been demonstrated to benefit ExPEC colonisation of the bladder via an interaction between the tip-associated YadC adhesin and Annexin A2 [34]. Furthermore, Yad fimbriae can bind to xylose-containing glycans and have also been implicated in the colonisation of intestinal epithelial cells by commensal *E. coli,* although a defined receptor interaction was not proposed [30]. While these studies provide molecular details of specific fimbriae-receptor interactions, they are not always exclusive, and chaperone-usher fimbriae can display flexibility in affinity for a variety of distinct glycan residues [43]. Allelic variation in the YadC adhesin observed in this study and by others [38], including within the predicted xylose binding pocket in the lectin domain, likely explain differences in receptor binding. Furthermore, given the complexities of host glycans and the number of distinct organs that could be encountered by circulating ExPEC during BSI, it is logical that fimbriae displaying a range of eukaryotic receptor affinities would benefit BSI progression.

There is also evidence that certain fimbriae can promote macrophage uptake and survival during phagocytosis [44]. This is also the case for Yad fimbriae, at least when tested *in vitro,* with several reports suggesting they benefit survival within cultured macrophages and even promote invasion of adhered epithelial cells [34,45]. The latter further supports a likely crucial role for Yad in tissue adhesion during UTI and BSI [30,34]. In contrast, expression of Yad fimbriae in enterohaemorrhagic *E. coli* was found to decrease adhesion to cultured epithelial cells potentially by interfering with other cell surface features [46]. However, a more recent study identified that Yad may provide enterohaemorrhagic *E. coli* a fitness benefit *in vivo* by promoting expression of the type 3 secretion system, a key virulence factor for this intestinal pathotype. Furthermore, this study showed that Yad was required for longer-term intestinal colonisation in Streptomycin-treated mice, suggesting they could also play an adhesive role within the gut niche [47]. Nevertheless, our data support a mounting body of evidence that Yad fimbriae play a more crucial role in extraintestinal niches. This potentially extends to natural animal hosts also, as it has been shown that Yad fimbriae from avian pathogenic *E. coli* contribute to infection of chickens by promoting adhesion and replication within multiple infected organs [45]. In line with these studies, our genomic analysis shows that the Yad fimbrial genes are highly prevalent across *E. coli* strains sourced from diverse ecological niches and exhibit significant sequence variation within the lectin domain, consistent with an adhesin that could interact with multiple different receptor binding targets.

The phase-variable regulation of Type 1 and P fimbriae is well characterised, with the former being under the control of an invertible promoter element and the latter being controlled by a combination of regulatory proteins and DNA methylation sites within its promoter region [22–24]. Yad fimbriae, on the other hand, are not phase-variable, with previous work linking Yad expression to environmental stimuli such as temperature and oxygen tension in non-pathogenic K-12 [30,35]. Furthermore, the *yad* locus is repressed by the nucleoid associated protein and global transcriptional regulator H-NS. H-NS functions by binding to AT-rich DNA sequences and silencing their transcription [48]. This often corresponds to horizontally-acquired foreign DNA, such as genomic islands that carry diverse virulence genes [49–51]. Many chaperone-usher fimbriae are encoded within genomic islands and are repressed by H-NS [13]. Counter-repression of H-NS is therefore thought to occur in response to host-specific cues [52]. For example, growth at human physiological temperature (37 °C) or greater is known to contribute to destabilisation of H-NS binding to DNA [48,53,54]. This destabilisation renders H-NS susceptible to the Lon protease *in vivo,* thus acting as a mechanism of de-repression within the host and a likely the reason why we have observed such high upregulation of *yad* genes within infected tissue [55]. Perhaps counterintuitively, Yad fimbriae were previously shown to exhibit higher expression at lower temperatures *in vitro* and under anaerobic conditions via a mechanism involving the oxygen-dependent transcription factor ArcA[30]. It was proposed that this would benefit formation of biofilms in the environment. However, ArcA does not contribute to *E. coli* fitness during BSI and our experiments were all performed at 37 °C with aeration [56]. Furthermore, the increased expression of Yad that we observe in response to human serum is a rapid switch, as opposed to a more stable niche such as a biofilm [30]. While we did not identify a precise mechanism of how Yad is regulated beyond H-NS de-repression, it is possible that a specific factor found in serum (such as a metabolite) is sensed by an ExPEC regulatory protein to fine tune *yad* expression. However, the chemical complexity of serum is vast and contains thousands of bioactive molecules, according to the serum metabolome database (https://www.serummetabolome.ca/statistics). This makes identification of such a signalling metabolite extremely difficult; however, we have narrowed down this idea by demonstrating that heat-inactivated serum does not reverse Yad upregulation, suggesting it is independent of complement activity. Furthermore, a recent integrative-omics study of sepsis-causing pathogens revealed that certain distinct clinical isolates of *E. coli* also display upregulation of *yad* in response to serum [57]. We hypothesise that such a regulatory protein would subsequently antagonise and outcompete destabilised H-NS for binding to the Yad region, as has been reported for other virulence factors such as the type 3 secretion system [58,59]. Our data therefore suggests that entry into the bloodstream acts as an acute trigger to promote expression of Yad fimbriae. This aligns with our previous work showing that expression of Type 1 and P fimbriae is also enhanced during BSI, and evokes a pathway for synergistic host-induced activation of fimbriae expression during ExPEC dissemination [25]. This model would also explain the partial decrease in ExPEC infection observed upon deletion of individual chaperone-usher loci and is consistent with the diversity of receptors encountered by ExPEC in the host, allowing for functional redundancy of fimbriae-mediated adherence. Such redundancy would also account for the lack of Yad fimbriae in phylogroup D *E. coli* (typically associated with extra-intestinal infections), where other adhesins or fimbriae may compensate [60].

The reasons underlying the ecological benefit of cellular adhesion during BSI remain to be fully elucidated given the apparent “dead-end” nature of such infections. We posit that adherence represents an intrinsic survival strategy for ExPEC to adapt to new environments, avoid immune clearance and successfully colonise the host. Understanding the molecular basis of fimbriae-receptor interactions also opens to door to new therapeutic possibilities. These include natural receptor antagonists, chemical small molecule inhibitors, and monoclonal antibodies [61–63]. Such approaches would address urgent needs in the face of rapidly rising rates of antibiotic resistance.

## Materials and methods

### Ethics statement

Animal experiments were performed in accordance with the Animals in Scientific Procedures Act (ASPA) of 1986 (personal project licence PP8850146 reviewed and approved by the United Kingdom Home Office). The procedures were subject to local ethical approval by the Animal Welfare and Ethical Review Board at Newcastle University with consideration given to the 3Rs principles where possible so that all efforts were made to minimise unnecessary animal suffering.

### Animal infection experiments

The BSI model was carried out as previously described [4,7,25]. Wild type and mutant strains were cultured overnight in LB broth, normalised to an OD_600_ of 0.4 in sterile PBS and mixed at a 1:1 ratio, making a final concentration of ~2 x 10^8^ CFU/ml. For complementation studies, 1:1 mixtures of wild type and mutant strains carrying either pSU-*yad* or pSU2718 as an empty vector control were prepared in the same manner. 100 µl of either mixture was used to inoculate 8-week old female BALB/c mice (Charles River) by administration directly into the tail vein (~10^7^ CFU). Mice were monitored for weight loss and adverse effects over 24 hours, before being euthanised by cervical dislocation. For quantification of colonisation, livers and spleens obtained post-mortem were transferred to tubes containing PBS and homogenised mechanically. Homogenates were serially diluted in PBS and replica spot-plated on LB agar plates with or without chloramphenicol in parallel, to determine the ratio of mutant to wild type cells in each organ. Competitive indices were calculated by dividing the ratio of mutant to wild type in organ homogenates by the same ratio in the inoculum. Statistical significance for competitive infections was determined using the Wilcoxon signed-rank test (hypothetical value of 0) on log-transformed values determined by the above calculation or by Wilcoxon matched-pairs signed-rank test on counts for each strain.

### Bacterial strains and growth conditions

Details of strains and mutant derivatives are listed in S2 Table. Overnight cultures were set up by inoculating single colonies into 5 ml LB broth. After growth overnight at 37 °C with shaking at 200 rpm, cultures were back-diluted by 100-fold into fresh LB broth, SOB or Minimal Essential Media with HEPES (MEM-HEPES; Sigma) for growth experiments. Growth was measured by reading optical density at 600 nm (OD_600_) using a FLUOstar OMEGA microplate reader (BMG Labtech). Where required, media was supplemented with antibiotics (Merck; 100 µg/mL ampicillin, 50 µg/mL kanamycin, 20 µg/mL chloramphenicol, 50 µg/mL Gentamicin) or the indicated volume of pooled human serum (Life Science Group).

### Generation of gene deletions by Lambda Red Recombineering

Plasmids pKD3 or pKD4 were used to amplify the FRT-chloramphenicol or FRT-kanamycin cassettes by Q5 High-Fidelity PCR (New England Biolabs) with primers containing 50 base flanks directly homologous to regions adjacent to the target gene [64]. Parental strains containing pKD46 were grown in SOB plus ampicillin for 2 hours at 30 °C before being spiked with 100 mM L-arabinose for an hour. Cells were pelleted and washed three times in ice-cold water before being resuspended at 100-fold concentration. Washed cells were transformed with ~500 ng of pKD3 derived PCR product by electroporation and recovered in SOC before being plated on LB agar with antibiotics at 37 °C. Successful gene deletions were identified by colony PCR using GoTaq G2 polymerase (Promega). Antibiotic resistance cassettes were removed using plasmid pCP20 by culturing mutant strains without selection at 42 °C. Primers used in this study are listed in S3 Table.

### Plasmid construction

Details of all plasmids used in this study are listed in S4 Table. To generate the transcriptional reporter fusion of the *yad* promoter to the *luxCDABE* operon in plasmid pMK1*lux*, ~ 200 bases upstream of the *yadN* coding region from CFT073 was amplified by PCR using Q5 polymerase (New England Biolabs) and primers flanked with 5’ EcoRI and 3’ BamHI restriction sites [65]. Plasmid pHNS-cm containing the *hns* gene from EC958 cloned into the pSU2718 expression vector has been described previously [37]. To generate the *yad* fimbriae complementation plasmid, the corresponding coding sequence from EC958 was amplified by PCR using Q5 polymerase (New England Biolabs) and ligated into HindIII digested plasmid pSU2718 by homologous alignment cloning as previously described [66]. Purified PCR products were digested with the corresponding enzymes and ligated into linearised plasmid backbones using T4 ligase (Promega). All plasmid constructs were verified by sequencing (Eurofins).

### Plasmid stability assay

For *in vivo* complementation studies, plasmid stability was first determined by inoculating single colonies of pSU2718 or pSU-*yad* transformed strains into LB media and incubating statically at 37°C for 24 hours. Mixtures were sub-cultured at a ratio of 1/1000 after 24 hours for three consecutive days. CFU counts were determined at day 0 before and after each 24-hour sub-culture on both plain LB and LB containing chloramphenicol, to determine the proportion of cells that retained pSU2718 in the absence of antibiotic.

### Serum killing assays

Overnight cultures of bacteria were normalised to an OD_600_ of 0.6. 15 µl of each culture was added to 85 µl of PBS in a flat-bottomed 96-well plate (Greiner). To these wells, 100µl of human serum was added at which point a sample was taken for serial dilution and CFU determination, representing T = 0. The mixtures were then incubated statically at 37°C for 90 minutes before CFU determination. Pooled human serum from healthy volunteers was purchased from Life Science Group Ltd. Where indicated, serum was inactivated by incubating at 56 °C for 30 minutes.

### LUX-promoter fusion transcriptional reporter assays

Reporter assays were carried out in white walled/clear flat-bottom microtiter plates (200 µL culture volume) using a FLUOstar OMEGA microplate reader (BMG Labtech). Plates were incubated at 37 °C with shaking. At regular intervals, absolute luminescence and OD_600_ were measured in tandem. Relative luminescence units (RLU) were calculated by dividing the luminescence by OD_600_ at each timepoint. Experiments were performed on three independent occasions.

### SDS-PAGE and Western blot analysis

Samples of bacterial culture were normalised by OD_600_ and centrifuged at 5000 rpm to remove the supernatant. Cell pellets were resuspended in 2x Laemmli buffer acidified with 3 µL of concentrated hydrochloric acid and boiled for 15 minutes. Samples were neutralised with saturated sodium hydroxide and separated by SDS-PAGE using 4–12% Bis-Tris NuPAGE gels (Invitrogen). Proteins were transferred to a 0.45 µM nitrocellulose membrane (GE Healthcare) using the XCell II Blot module (Invitrogen). Membranes were blocked with a 5% skim-milk solution for one hour before being washed with PBS-Tween and incubated overnight with a 1% skim-milk solution containing YadN-specific antibodies at a concentration of 1/100. Membranes were washed three times with PBS-Tween and incubated with anti-rabbit HRP-conjugated secondary antibodies (1/1000) for one hour. Western blots were finally incubated with SuperSignal West Pico chemiluminescent substrate (Pierce) for five minutes before imaging using a G:Box Chemi system (Syngene).

### Whole cell ELISA

To detect Yad cell surface expression, ExPEC cells were cultured in MEM-HEPES spiked with 50% human serum or sterile PBS as a control until mid-log phase (OD_600_ = ~0.5) and concentrated to an OD_600_ of 1.0 in 100 mM sodium carbonate buffer (pH 9.5). MaxiSorp 96-well ELISA plates (Thermo Fisher) were coated with 100 µL of cells per well overnight at 4 °C. Coated wells were blocked 5% skim milk solution in PBS-Tween (0.05%) for 1 hour at room temperature. Wells were then probed with 100 µL of anti-YadN antibody (1/100 dilution in PBS-Tween) for 1 hour before 3 washes using 250 µL PBS-Tween. The secondary antibody used was AP-conjugated anti-rabbit IgG (Sigma) at a concentration of 1/10,000 and incubated for 1 hour prior 3 further washes. The reaction was developed using 100 µL of pNPP substrate (Sigma) for 30 minutes and absorbance was measured at 420 nm.

### RNA extraction, cDNA generation and quantitative reverse transcription PCR

Bacterial cultures grown in MEM-HEPES until mid-log phase (OD_600_ = ~0.5) were spiked with 50% human serum or sterile PBS as a control for 20 minutes, before being mixed with 2 volumes of RNA protect (Qiagen) for 15 minutes at room temperature prior to centrifugation to remove the supernatant. Total RNA was extracted using the Monarch Total RNA Miniprep kit (New England Biolabs). For *in vivo* samples, ~ 10mm section of infected hepatic or splenic tissue was immediately removed post-mortem and were stored in RNA*later* (Ambion) for 24 hours at 4 °C. The tissue samples were then homogenised using a TissueLyser LT (Qiagen) and total RNA was extracted using the mirVANA kit (Ambion). Samples were DNase treated using DNaseI (Promega) and integrity was assessed by Qubit analysis (Thermo Fisher).

Normalised RNA samples were used to make cDNA using the LunaScript RT SuperMix kit (New England Biolabs). qRT-PCR was performed in a CFX Duet Real Time PCR machine (Bio Rad) using Luna Universal qPCR master mix (New England Biolabs). Each reaction was performed in technical triplicate, averaged and used as one biological replicate. The housekeeping gene *groEL* was used for normalisation and data analysis by the 2^-ΔΔCT^ method. Primer sequences are listed in S3 Table.

### Bioinformatic analysis of Yad carriage

Strains containing *yad* operons were identified from a collection of 1310 completely sequenced, MLST- and phylogroup-typeable *E. coli* strains generated from NCBI RefSeq (accessed 20/01/2021) using *htrE*_EC958_ (EC958_RS00785) as a tBLASTn query with an 80% alignment length and identity threshold [67,68]. The prevalence of each *yad* operon-associated gene was quantified using the EC958 Yad amino acid sequences (EC958_RS00795 to EC958_RS00765) as a tBLASTn query at an 80% alignment length threshold. *E. coli* MLST was performed using the MLST tool (https://github.com/tseemann/mlst) and the PubMLST database, with phylogroup being inferred from MLST information [69,70]. Yad prevalence across source niches was interrogated on a collection of ~10,000 *E. coli* draft genomes from the top 100 sequence types (STs) in Enterobase, where source niche metadata was available (downloaded 18/12/2020; referred to as the 100ST database [71–73]. Complete YadC (adhesin) sequences were aligned and subsequently utilised to generate a maximum likelihood phylogeny using CLC Main Workbench 23.0.4 (QIAGEN) with the neighbour joining construction method, the WAG protein substitution model, and 100 bootstraps. The resulting phylogenetic tree was visualised using the interactive Tree of Life as a midpoint rooted tree [74]. The predicted structure of YadC_EC958_ was generated using ColabFold with default parameters [75]. Amino acid conservation was quantified with the R package bio3d (v2.4-5)using similarity scoring (BLOSUM62 matrix) and mapped onto the predicted structure with ChimeraX (v1.10.1) [76,77]. The amino acid identities of the YadC lectin domain and pilin domain were generated using YadC_EC958_ as a BLASTp query at an 80% length threshold.

### *In silico* docking of D-xylose to YadC

Models of K-12, EC958, and CFT073 YadC-LD (signal peptides removed) were generated using AlphaFold2 [78,79]. Binding pockets were predicted with FPocket and visualised in ChimeraX [77, 80–82]. Conserved binding pockets were used to define docking coordinates via RDKit on Galaxy [83,84]. D-Xylose (XLS) obtained from RCSB PDB and the YadC-LD models were processed using the tools Prepare ligand and Prepare receptor respectively in Galaxy [83,85,86]. Molecular docking was performed using VINA docking in Galaxy, ‌‌and the results with the highest conservation in docking sites and binding affinities were visualised in ChimeraX [77,82,87,88].

### Statistical analysis

Graphs and statistics were generated using GraphPad Prism software version 10. Statistical tests used are indicated throughout the manuscript and in the figure legends. *P* values of less than or equal to 0.05 were considered statistically significant.

## Supporting information

S1 Fig ExPEC strains are resistant to the bactericidal effects of human serum. Enumeration of ExPEC strains CFT073 and EC958 after a 90-minute exposure to 50% human serum (red) or heat-inactivated serum (HIS; grey) as a negative control. The *E. coli* K-12 strain MG1655, which is serum susceptible, was used as a positive control for serum killing. Data indicates the mean of 3 biological replicates.
(TIFF)

S2 Fig Prevalence of Yad fimbriae across *E. coli* of different ecological niches. Yad operon prevalence amongst the 100 sequence type draft *E. coli* genomes by source niche, where data is available. Strains containing the *htrE* usher at an 80% alignment length and identity threshold in a tBLASTn search (HtrE^EC958^ query) were considered Yad positive.
(TIFF)

S3 Fig Alignment of Yad protein sequences from reference UPEC strains EC958 and CFT073. Alignments of (A) YadN; (B) EcpD; (C) HtrE; (D) YadM; (E) YadL; (F) YadK; (G) YadC sequences performed in CLC Main Workbench v23.0.4.
(TIFF)

S4 Fig Modelling of YadC interaction with the ligand D-xylose. *In silico* analysis of D-xylose docked into surface hydrophobicity models of YadC-LD using AutoDock Vina and visualised with ChimeraX. Residues contacting D-xylose are labelled and ChimeraX-calculated contact points (default parameters) between D-xylose and the YadC-LD are shown as green dashed lines. Binding affinity estimated usingVina scoring in kcal/mol is shown. (A) MG1655 YadC-LD. (B) EC958 YadC-LD. (C) CFT073 YadC-LD.
(TIFF)

S5 Fig Transcriptional repression of the *yad* locus by H-NS in ExPEC strains. qRT-PCR analysis of the *yad* locus genes from CFT073 (light grey) and a corresponding Δ*hns* mutant (dark grey) in the top panel (A), or EC958 and a corresponding Δ*hns* mutant in the bottom panel (B). Cells were cultured in MEM-HEPES prior to RNA extraction and cDNA conversion. The bars depict the relative fold change above the wild type expression levels and the error bars represent the standard deviation (n = 3). ** and *** indicate *P* < 0.05 and *P* < 0.01, respectively, as determined by a two-tailed t-test.
(TIFF)

S6 Fig Yad cell-surface expression in ExPEC strain EC958. Whole cell ELISA detection of YadN cell-surface expression in EC958, Δ*hns* or Δ*yad* cultured in MEM-HEPES (grey bars) or MEM-HEPES supplemented with 50% human serum (HS; red bars). The error bars represent the standard deviation (n = 3).
(TIFF)

S7 Fig Deletion of the Yad locus does not impact ExPEC growth. Growth curves depicting optical density (600 nm) measurements taken during culture in MEM-HEPES. The left and right panels indicate data for CFT073 and EC958 plus their Δ*yad* mutant derivatives respectively. The error bars represent the standard deviation (n = 3).
(TIFF)

S8 Fig Validation of Yad cell-surface expression from pSU-*yad.* Whole cell ELISA detection of YadN cell-surface expression in CFT073 transformed with either empty pSU2718 or pSU-*yad.* Cells were cultured in MEM-HEPES and the error bars represent the standard deviation (n = 3).
(TIFF)

S9 Fig Stability of plasmid pSU2418 and its derivatives without selection. Enumeration of CFT073Δ*yad* transformed with pSU-*yad* at the indicated timepoints after sequential subculturing in LB media lacking antibiotic selection. Serial dilutions were plated on plain LB agar and LB agar containing chloramphenicol to calculate the percentage of the population that stably retained plasmid pSU-*yad.* The experiment was performed on two independent occasions.
(TIFF)

S1 Table Nucleotide identities of yad genes against the EC958 reference in yad positive strains. (CSV)

S2 Table Bacterial strains used in this study. (DOCX)

S3 Table Primers used in this study. (DOCX)

S4 Table Plasmids used in this study. (DOCX)

S1 File Raw data for all figures. (XLSX)
