# Supplementary figures and images for: Yad fimbriae are triggered by host cues and enhance extraintestinal pathogenic Escherichia coli tissue colonisation during bloodstream infection
Source: PLoS Pathog. 2026 Jun 1;22(6):e1014299. doi: 10.1371/journal.ppat.1014299 (PMC13245861; doi:10.1371/journal.ppat.1014299)

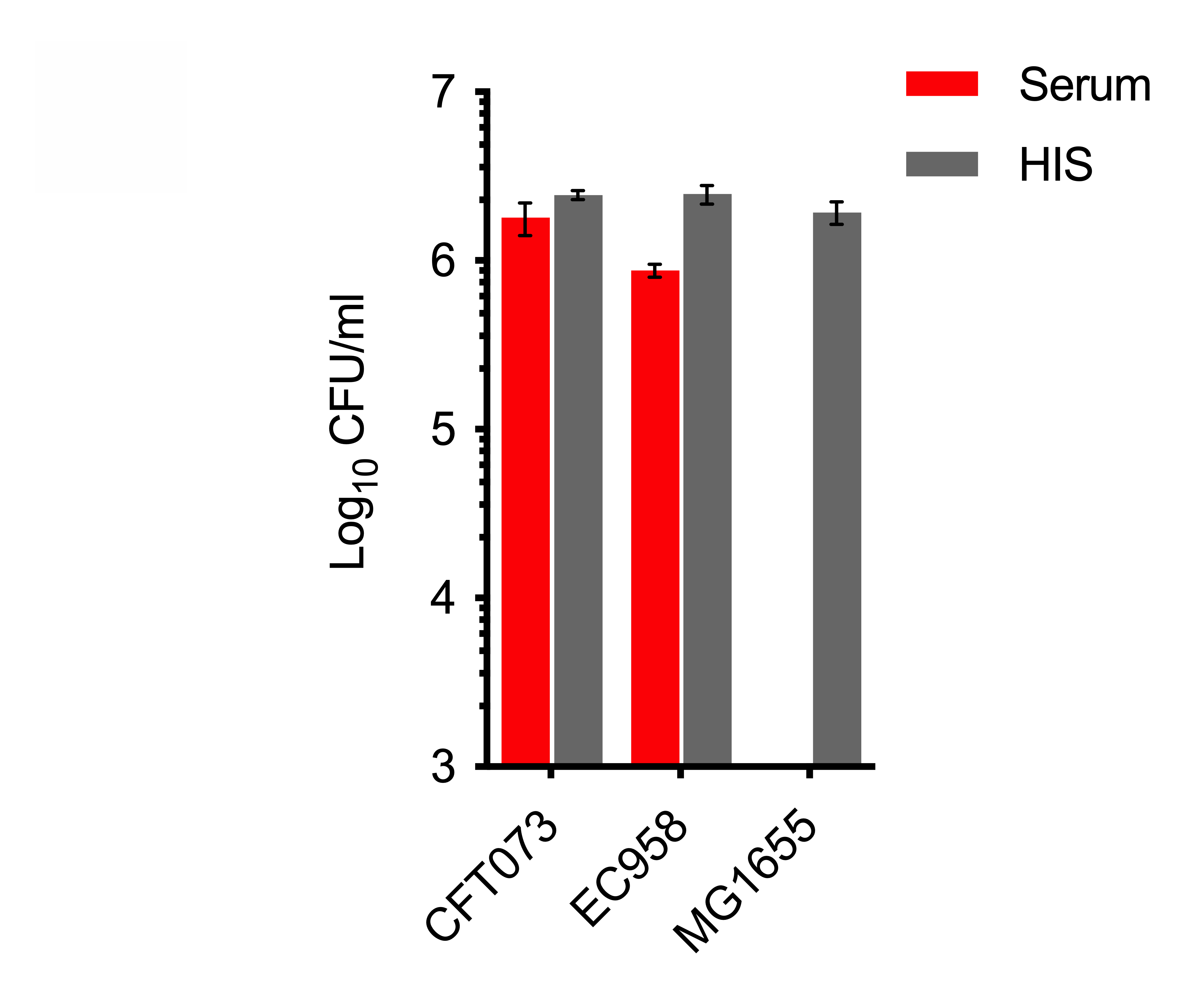

Supplement: S1 Fig — Enumeration of ExPEC strains CFT073 and EC958 after a 90-minute exposure to 50% human serum (red) or heat-inactivated serum (HIS; grey) as a negative control. The E. coli K-12 strain MG1655, which is serum susceptible, was used as a positive control for serum killing. Data indicates the mean of 3 biological replicates. (TIFF) [file ppat.1014299.s001.tiff]

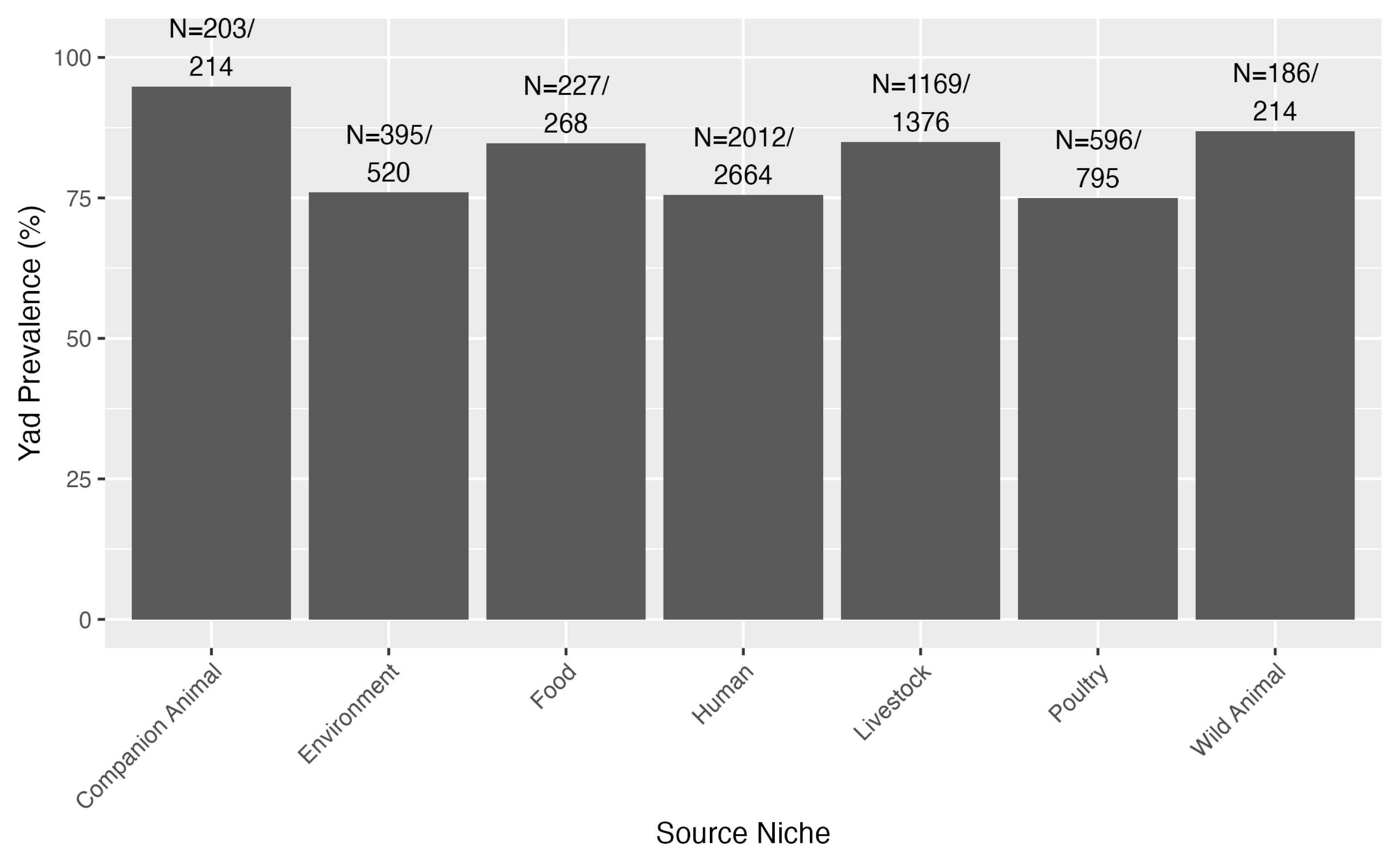

Supplement: S2 Fig — Yad operon prevalence amongst the 100 sequence type draft E. coli genomes by source niche, where data is available. Strains containing the htrE usher at an 80% alignment length and identity threshold in a tBLASTn search (HtrEEC958 query) were considered Yad positive. (TIFF) [file ppat.1014299.s002.tiff]

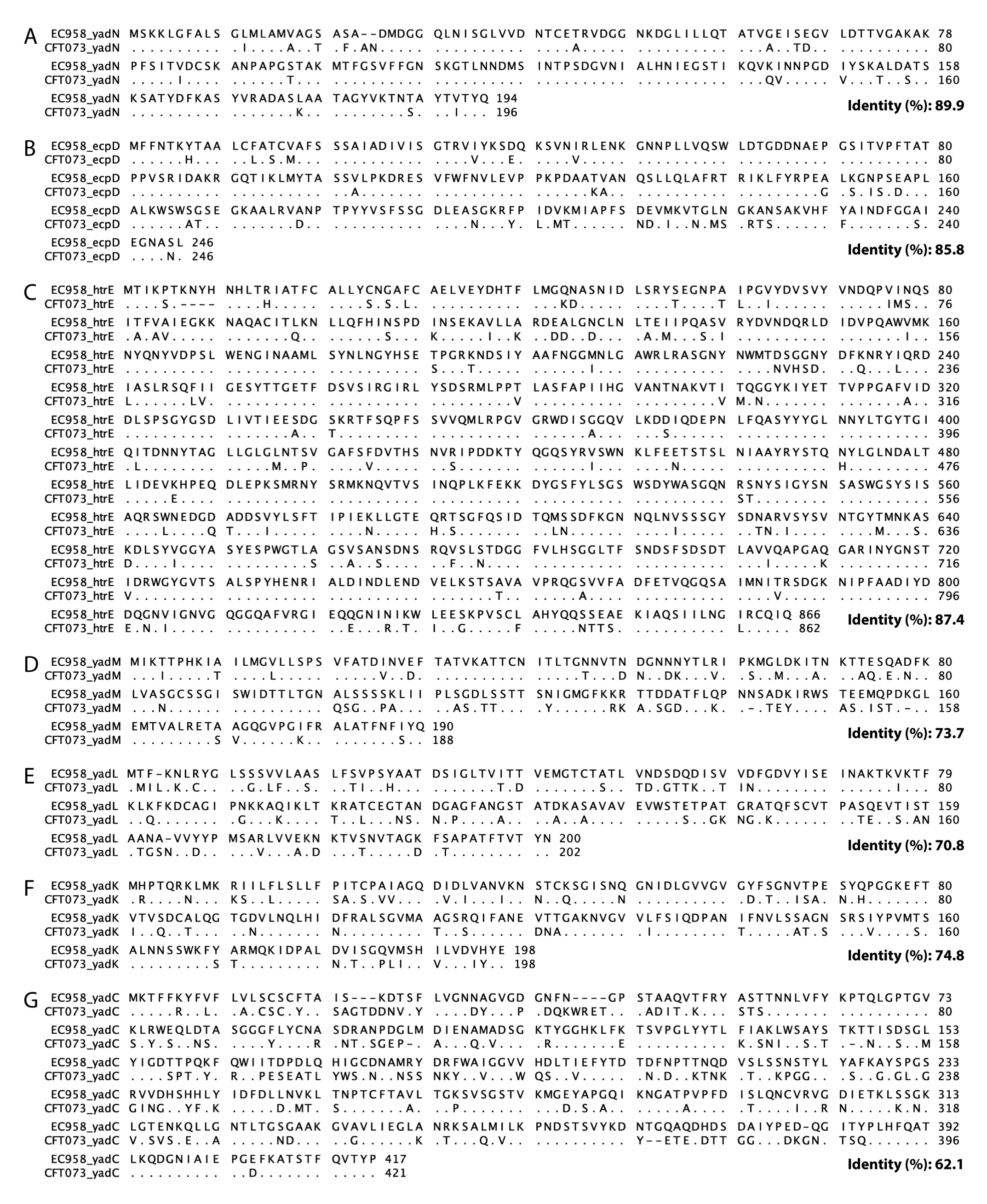

Supplement: S3 Fig — Alignments of (A) YadN; (B) EcpD; (C) HtrE; (D) YadM; (E) YadL; (F) YadK; (G) YadC sequences performed in CLC Main Workbench v23.0.4. (TIFF) [file ppat.1014299.s003.tiff]

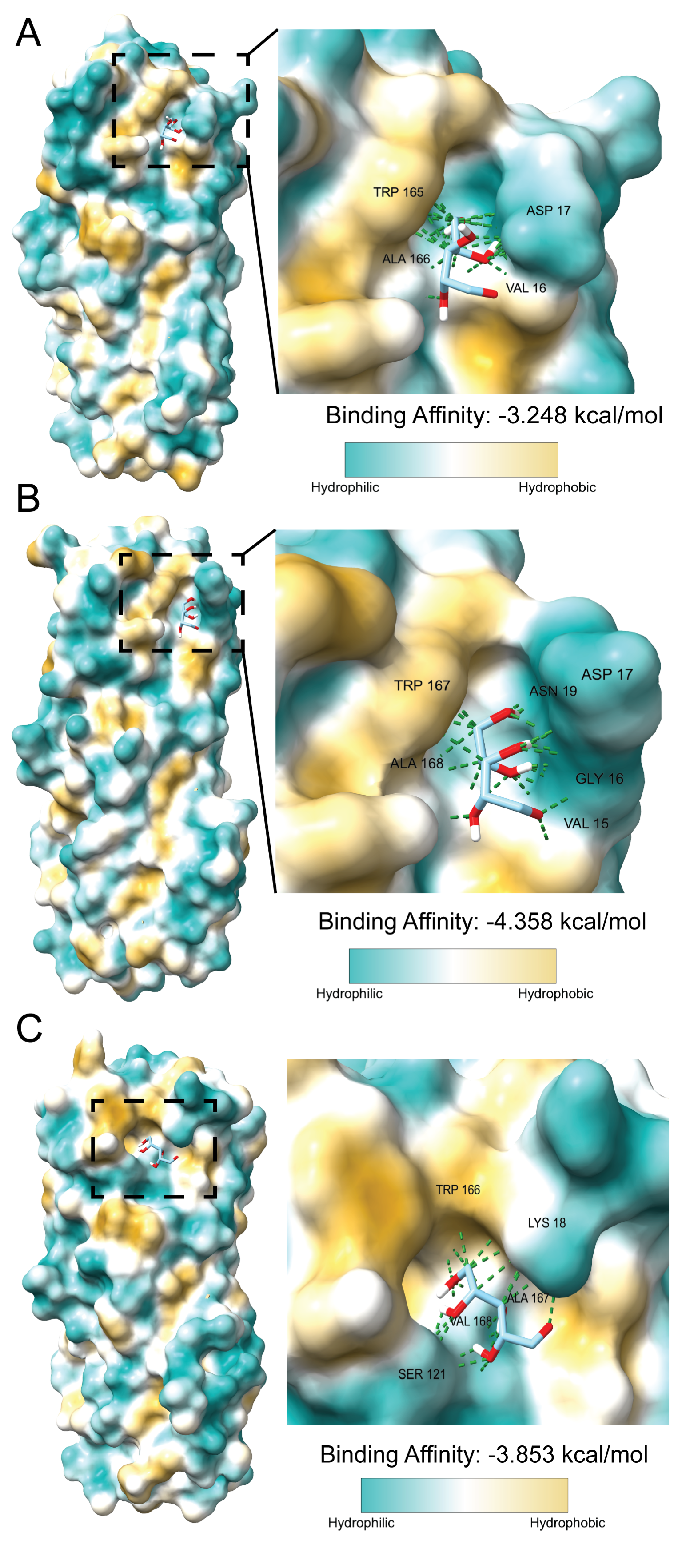

Supplement: S4 Fig — In silico analysis of D-xylose docked into surface hydrophobicity models of YadC-LD using AutoDock Vina and visualised with ChimeraX. Residues contacting D-xylose are labelled and ChimeraX-calculated contact points (default parameters) between D-xylose and the YadC-LD are shown as green dashed lines. Binding affinity estimated usingVina scoring in kcal/mol is shown. (A) MG1655 YadC-LD. (B) EC958 YadC-LD. (C) CFT073 YadC-LD. (TIFF) [file ppat.1014299.s004.tiff]

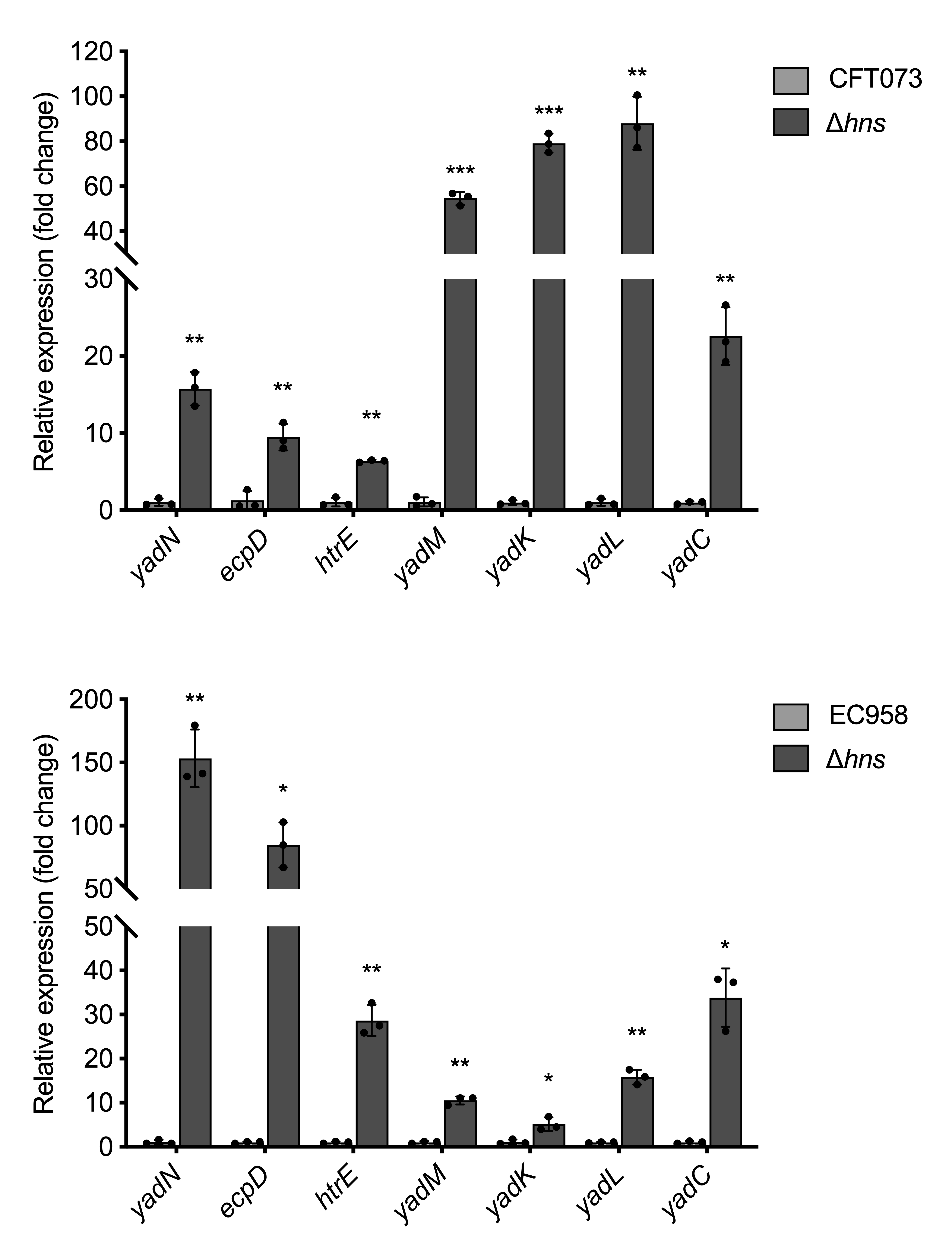

Supplement: S5 Fig — qRT-PCR analysis of the yad locus genes from CFT073 (light grey) and a corresponding Δhns mutant (dark grey) in the top panel (A), or EC958 and a corresponding Δhns mutant in the bottom panel (B). Cells were cultured in MEM-HEPES prior to RNA extraction and cDNA conversion. The bars depict the relative fold change above the wild type expression levels and the error bars represent the standard deviation (n = 3). ** and *** indicate P < 0.05 and P < 0.01, respectively, as determined by a two-tailed t-test. (TIFF) [file ppat.1014299.s005.tiff]

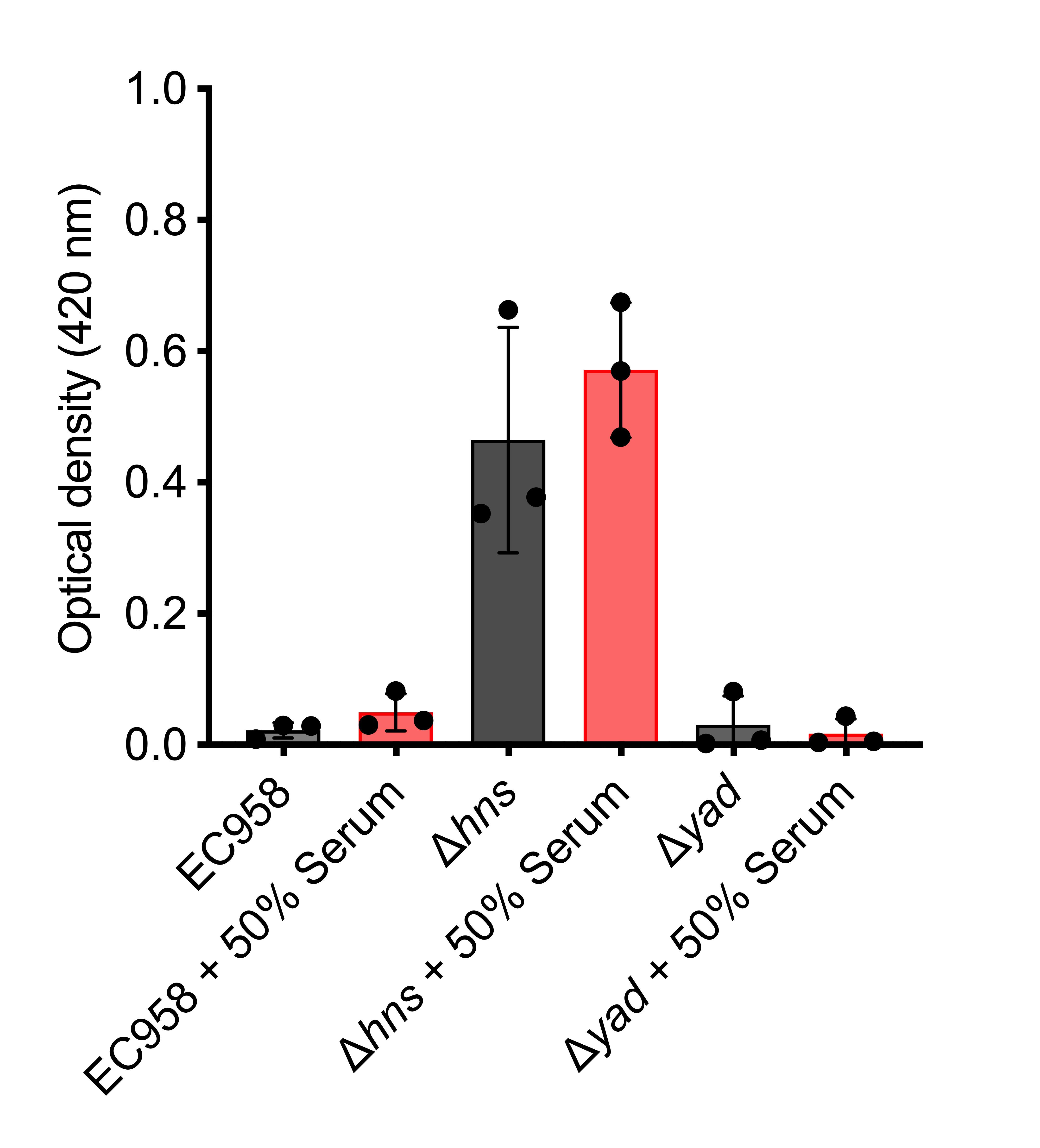

Supplement: S6 Fig — Whole cell ELISA detection of YadN cell-surface expression in EC958, Δhns or Δyad cultured in MEM-HEPES (grey bars) or MEM-HEPES supplemented with 50% human serum (HS; red bars). The error bars represent the standard deviation (n = 3). (TIFF) [file ppat.1014299.s006.tiff]

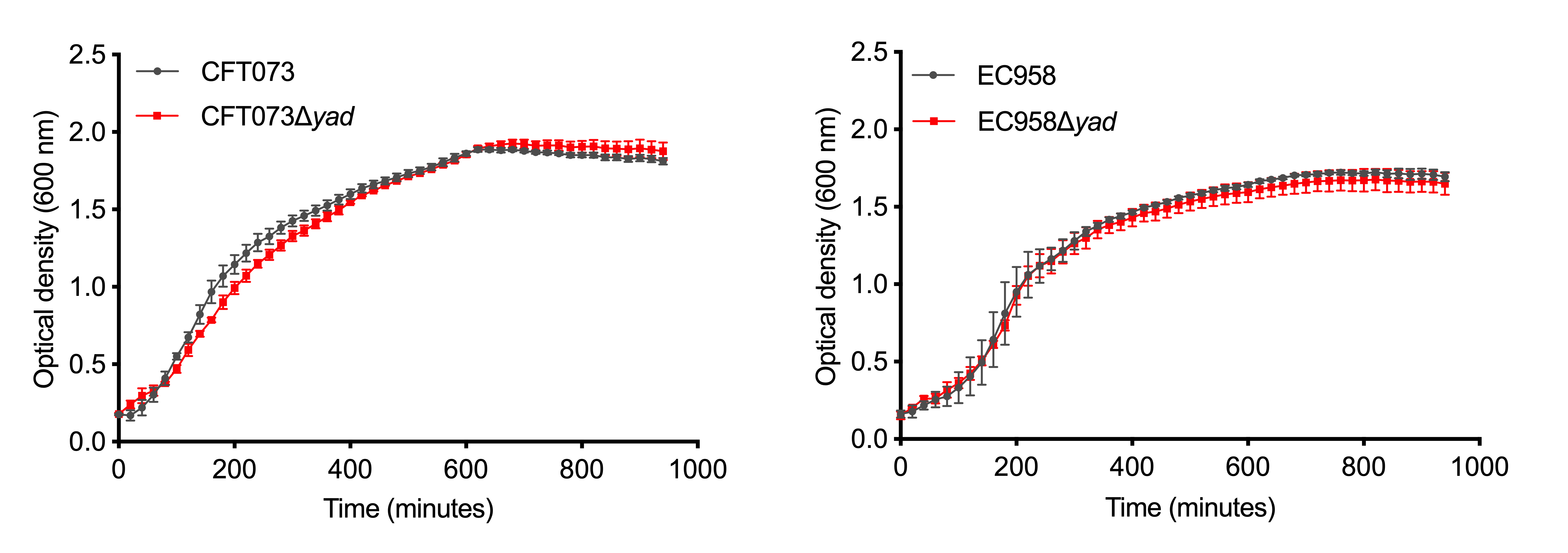

Supplement: S7 Fig — Growth curves depicting optical density (600 nm) measurements taken during culture in MEM-HEPES. The left and right panels indicate data for CFT073 and EC958 plus their Δyad mutant derivatives respectively. The error bars represent the standard deviation (n = 3). (TIFF) [file ppat.1014299.s007.tiff]

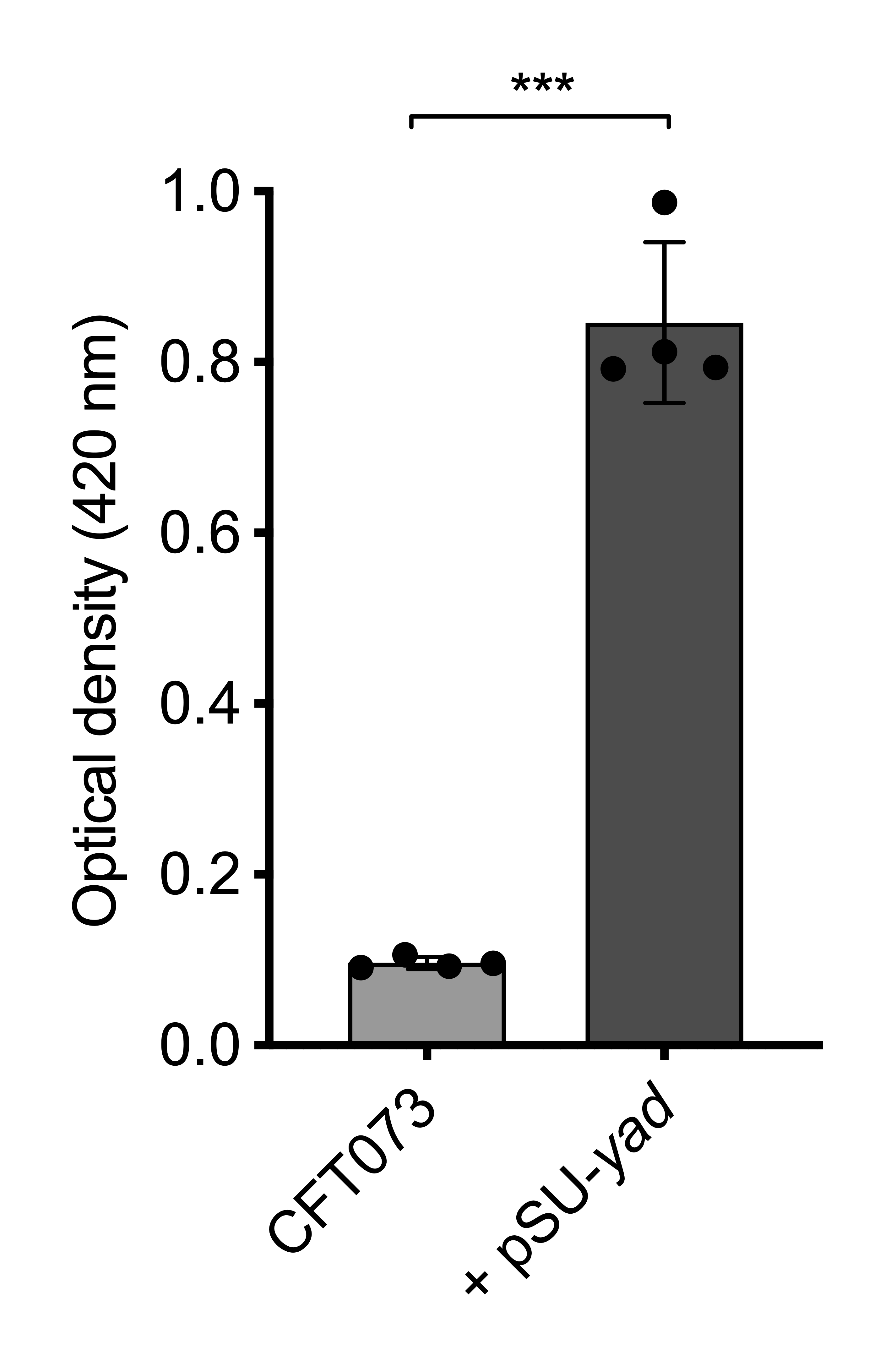

Supplement: S8 Fig — Whole cell ELISA detection of YadN cell-surface expression in CFT073 transformed with either empty pSU2718 or pSU-yad. Cells were cultured in MEM-HEPES and the error bars represent the standard deviation (n = 3). (TIFF) [file ppat.1014299.s008.tiff]

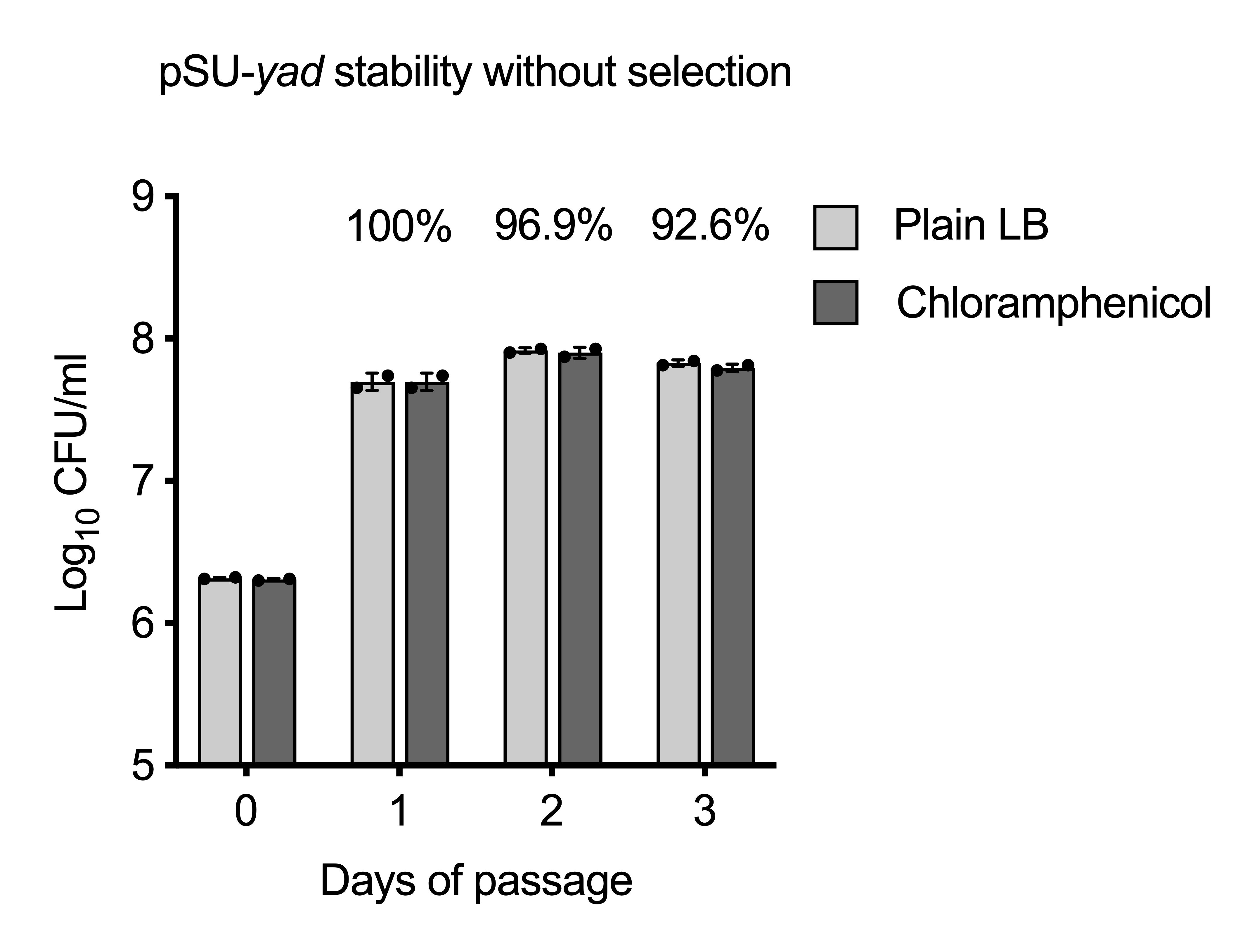

Supplement: S9 Fig — Enumeration of CFT073Δyad transformed with pSU-yad at the indicated timepoints after sequential subculturing in LB media lacking antibiotic selection. Serial dilutions were plated on plain LB agar and LB agar containing chloramphenicol to calculate the percentage of the population that stably retained plasmid pSU-yad. The experiment was performed on two independent occasions. (TIFF) [file ppat.1014299.s009.tiff]
