# Supplementary material for: Yad fimbriae are triggered by host cues and enhance extraintestinal pathogenic Escherichia coli tissue colonisation during bloodstream infection
Source: PLoS Pathog. 2026 Jun 1;22(6):e1014299. doi: 10.1371/journal.ppat.1014299 (PMC13245861; doi:10.1371/journal.ppat.1014299)
Supplement: S2 Table — (DOCX) [file ppat.1014299.s011.docx]

**S2 Table** – Bacterial strains used in this study.

| **Strain** | **Description** | **Source** |
| --- | --- | --- |
| CFT073 | Wild type *E. coli* strain CFT073; O6:K2:H1 serotype | Welch *et al.* 2002 *PNAS* 99(26):17020-4 |
| EC958 | Wild type *E. coli* strain EC958; O25b:H4 serotype | Totsika *et al.* 2011 *PLoS ONE* 6(10):e26578 |
| MG1655 | *E. coli* strain MG1655; OR: H48:K- serotype | Lab collection |
| CFT073∆*hns* | CFT073 *hns* deletion | This study |
| EC958∆*hns* | CFT073 *hns* deletion | This study |
| CFT073∆*yad* | CFT073 *yadN-ecpD-htrE-yadMLKC* locus deletion | This study |
| CFT073∆*yad* | EC958 *yadN-ecpD-htrE-yadMLKC* locus deletion | This study |
| ∆*yadN* | CFT073 *hns* deletion; Chl^R^ | This study |
| ∆*hns/yadN* | CFT073 *hns* and *yadN* double deletion; Kan^R^ Chl^R^ | This study |
