# Supplementary material for: Yad fimbriae are triggered by host cues and enhance extraintestinal pathogenic Escherichia coli tissue colonisation during bloodstream infection
Source: PLoS Pathog. 2026 Jun 1;22(6):e1014299. doi: 10.1371/journal.ppat.1014299 (PMC13245861; doi:10.1371/journal.ppat.1014299)
Supplement: S3 Table — (DOCX) [file ppat.1014299.s012.docx]

**S3 Table** – Primers used in this study.

| **Primer Name** | **Description** | **Sequence** |
| --- | --- | --- |
| *hns*_LRed_Fwd | *hns* KO forward primer | TCTATTATTACCTCAACAAACCACCCCAATATAAGTTTGAGATTACTACAGTGTAGGCTGGAGCTGCTTC |
| *hns*_LRed_Rev | *hns* KO reverse primer | AAAAAATCCCGCCGATGGCGGGATTTTTAGCATGTGCAATCTACAAAAGACATATGAATATCCTCCTTAG |
| *hns*_Check_Fwd | *hns* KO check forward primer | ATATGCCGCGTCTTTTCTGG |
| *hns*_Check_Rev | *hns* KO check reverse primer | GGCTTGAAGAAGAGATGGGC |
| *yadN*_LRed_Fwd | *yadN* KO forward primer | CATGGCATTGCATCAAAATCTCATCATCATGCAATGCTGTTATCTGCTTTGTGTAGGCTGGAGCTGCTTC |
| *yadN*_LRed_Rev | *yadN* KO reverse primer | TGATGTTATTCCCTGTATATATTCACCCGATCAATTTAAGCGGTTGATATCATATGAATATCCTCCTTAG |
| *yadN*_Check_Fwd | *yadN* KO check forward primer | ATTCACCCGATCAATTTAAGCG |
| *yadN*_Check_Rev | *yadN* KO check reverse primer | CGTTCAGTTACATGGCATTGC |
| *yadCFT073*_LRed_Fwd | *yadNMLKCecpDhtrE* KO CFT073 forward primer | CATCATGCAATGCTGTTATCTGCTTTATGGAATATTACTCAAGGATTTTTGTGTAGGCTGGAGCTGCTTC |
| *yadCFT073*_LRed_Rev | *yadNMLKCecpDhtrE* KO CFT073 reverse primer | GGCAATGATTTATCCGTATCGCACCGCCGTTGTCTGGCGGTGCGATAGTGCATATGAATATCCTCCTTAG |
| *yadCFT073*_Check_Fwd | *yadNMLKCecpDhtrE* KO CFT073 check forward primer | CGTTCAGTTACATGGCATTGC |
| *yadCFT073*_Check_Rev | *yadNMLKCecpDhtrE* KO CFT073 check reverse primer | ATGATGTTGTGCTGTCTCGC |
| *yadEC958*_LRed_Fwd | *yadNMLKCecpDhtrE* KO EC958 forward primer | TTCCTCTATCGGGAATCCCTGCCTGAAAGGGCCCAATTGTAGTTAAACTCGTGTAGGCTGGAGCTGCTTC |
| *yadEC958*_LRed_Rev | *yadNMLKCecpDhtrE* KO EC958 reverse primer | GCAATGATTTATCTATTGTGGCACCGCCATTACCAGACGGTGCCACAATGCATATGAATATCCTCCTTAG |
| *yadEC958*_Check_Fwd | *yadNMLKCecpDhtrE* KO EC958 check forward primer | CTGAAAGGGCCCAATTGTAGT |
| *yadEC958*_Check_Rev | *yadNMLKCecpDhtrE* KO EC958 check reverse primer | GTAATGTCGTGCTGTCTGGC |
| pMK1*lux*-P*_yadN_*_Fwd | Forward primer for cloning *yadN* promoter with EcoRI | CCCGAATTCCTTAATTTTTTAAAGTTAATTTTAC |
| pMK1*lux*-P*_yadN_*_Rev | Reverse primer for cloning *yadN* promoter with BamHI | GGATCCAAAGCAGATAACAGCATTGCATGATG |
| pMK1*lux*_Check_Fwd | Forward primer to check pMK1*lux* cloning | CTATAAAAATAGGCGTATCAC |
| pMK1*lux*_Check_Rev | Reverse primer to check pMK1*lux* cloning | CTGGCCGTTAATAATGAATG |
| pSU-*yad_*Fwd | Forward primer for cloning *yad* with *Hind*III | GACCTGCAGGCATGCAAGCTATGAAGGAGGTCGTTATGTCTAAAAAATTAGGTTTTGC |
| pSU-*yad_*Rev | Reverse primer for cloning *yad* with *Hind*III | CGACGGCCAGTGCCAAGCTTCAGGGATAGGTTACCTGG |
| *yadN_CFT_*qPCR_Fwd | Forward primer qRT-PCR | TTAGCGGTCTGGTCGTTGAT |
| *yadN_CFT_*qPCR_Rev | Reverse primer qRT-PCR | GGTTTGCAGCAGGATCAGAC |
| *ecpD_CFT_*qPCR_Fwd | Forward primer qRT-PCR | AACTGTTCTATCGCCCGGAA |
| *ecpD_CFT_*qPCR_Rev | Reverse primer qRT-PCR | TTTGCCTTCAGATGTTGCCC |
| *htrE_CFT_*qPCR_Fwd | Forward primer qRT-PCR | GTGAATCTTACACGACCGGC |
| *htrE_CFT_*qPCR_Rev | Reverse primer qRT-PCR | TTGGCGCAAAACTGGCTAAT |
| *yadM_CFT_*qPCR_Fwd | Forward primer qRT-PCR | CATACGCTGGAGTGCATCAG |
| *yadM_CFT_*qPCR_Rev | Reverse primer qRT-PCR | ACACCTTGCCCTACACTTGT |
| *yadL_CFT_*qPCR_Fwd | Forward primer qRT-PCR | CGATTACCCAATGAGTGCGG |
| *yadL_CFT_*qPCR_Rev | Reverse primer qRT-PCR | AAGTCACGGTGAATGTTGCC |
| *yadK_CFT_*qPCR_Fwd | Forward primer qRT-PCR | ATCCGGTCATGTCATCAGCA |
| *yadK_CFT_*qPCR_Rev | Reverse primer qRT-PCR | CACATGGCTTATCAGTGGGC |
| *yadC_CFT_*qPCR_Fwd | Forward primer qRT-PCR | ATCGGCGATTCTCCAACTCA |
| *yadC_CFT_*qPCR_Rev | Reverse primer qRT-PCR | CCACCTACGGCCCAGTATTT |
| *yadN_EC958_*qPCR_Fwd | Forward primer qRT-PCR | TCTGCAGATATGGACGGTGG |
| *yadN_EC958_*qPCR_Rev | Reverse primer qRT-PCR | AGCAGGATCAGGCCATCTTT |
| *ecpD_EC958_*qPCR_Fwd | Forward primer qRT-PCR | AAGTGTCAACATTCGCCTGG |
| *ecpD_EC958_*qPCR_Rev | Reverse primer qRT-PCR | CCAGGCTCAGCATTGTCATC |
| *htrE_EC958_*qPCR_Fwd | Forward primer qRT-PCR | TGGTGCTTTCTGTGCTGAAC |
| *htrE_EC958_*qPCR_Rev | Reverse primer qRT-PCR | TCGTACACACCCGGTATAGC |
| *yadM_EC958_*qPCR_Fwd | Forward primer qRT-PCR | GGGCCTGGATAAGATCACGA |
| *yadM_EC958_*qPCR_Rev | Reverse primer qRT-PCR | CGTTTCCGGTCAGAGTGGTA |
| *yadL_EC958_*qPCR_Fwd | Forward primer qRT-PCR | TTAACCAAGCGAGCCACATG |
| *yadL_EC958_*qPCR_Rev | Reverse primer qRT-PCR | GTTTCGGTGCTCCAGACTTC |
| *yadK_EC958_*qPCR_Fwd | Forward primer qRT-PCR | TTCCCATTACCTGTCCAGCG |
| *yadK_EC958_*qPCR_Rev | Reverse primer qRT-PCR | CCCTGGTTACTGATTCCGCT |
| *yadC_EC958_*qPCR_Fwd | Forward primer qRT-PCR | ATGGTAATTTCAACGGCCCG |
| *yadC_EC958_*qPCR_Rev | Reverse primer qRT-PCR | TGTCGGGCCAAGTTGAGTAG |
| *focA_CFT_*qPCR_Fwd | Forward primer qRT-PCR | GTCTGCTGTCACCACGGTTA |
| *focA_CFT_*qPCR_Rev | Reverse primer qRT-PCR | CGAACCTGTCCAAGATTAACCG |
| *fimA_CFT_*qPCR_Fwd | Forward primer qRT-PCR | TTGTTCTGTCGGCTCTGTCC |
| *fimA_CFT_*qPCR_Rev | Reverse primer qRT-PCR | AAGCGGCGTTAACAACTTCC |
| *yehA_CFT_*qPCR_Fwd | Forward primer qRT-PCR | TTTAAAAGTGTGACTGGCGTACC |
| *yehA_CFT_*qPCR_Rev | Reverse primer qRT-PCR | ACCAACAGCAACACGATCCT |
| *c1935_CFT_*qPCR_Fwd | Forward primer qRT-PCR | GCAATATAACGAGCCTGGAACG |
| *c1935_CFT_*qPCR_Rev | Reverse primer qRT-PCR | TTGGACAGTACAGGGGAGGT |
| *yfcO_CFT_*qPCR_Fwd | Forward primer qRT-PCR | TGTCAGTGATGGGATGCCTG |
| *yfcO_CFT_*qPCR_Rev | Reverse primer qRT-PCR | TGGGTTGCGCAAATGATCAC |
| *ygiL_CFT_*qPCR_Fwd | Forward primer qRT-PCR | AACCTCGGTGAAGTTGCTGA |
| *ygiL_CFT_*qPCR_Rev | Reverse primer qRT-PCR | AACAGTGTTAGTGCCGTCAGA |
| *papA_CFT_*qPCR_Fwd | Forward primer qRT-PCR | CGCACCATCAAATTTTACGCG |
| *papA_CFT_*qPCR_Rev | Reverse primer qRT-PCR | CAAGTGGTCCGCAGAGTGA |
| *papA2_CFT_*qPCR_Fwd | Forward primer qRT-PCR | AGGCACCTTCAGCTACATTCT |
| *papA2_CFT_*qPCR_Rev | Reverse primer qRT-PCR | GAGACCGGGGCTTCAAATCT |
| *aufA_CFT_*qPCR_Fwd | Forward primer qRT-PCR | ATTGTATAAGCGGCGGTGGA |
| *aufA_CFT_*qPCR_Rev | Reverse primer qRT-PCR | CGACTGGTGTAGGGATTCGAA |
| *fimA_EC_*qPCR_Fwd | Forward primer qRT-PCR | TTGTTCTGTCGGCTCTGTCC |
| *fimA_EC_*qPCR _Rev | Reverse primer qRT-PCR | AAGCGGCGTTAACAACTTCC |
| *yadN_EC_*qPCR_Fwd | Forward primer qRT-PCR | TGCGGATTTGGAAGTTGCATC |
| *yadN_EC_*qPCR _Rev | Reverse primer qRT-PCR | TGACGGTGTTAATATTGCTCTGC |
| *yfcO_EC_*qPCR_Fwd | Forward primer qRT-PCR | ACCGGCGTTACTGTGACTTT |
| *yfcO_EC_*qPCR _Rev | Reverse primer qRT-PCR | GATCGTGGCGCTTATTGTCG |
| *yehA_EC_*qPCR_Fwd | Forward primer qRT-PCR | TCCAGTGCGCCAGATTTAACT |
| *yehA_EC_*qPCR_Rev | Reverse primer qRT-PCR | AGAAATTGCGGCAAAGTCAAAAC |
| *1770_EC_*qPCR_Fwd | Forward primer qRT-PCR | TTGTTGCCGATACTTGCGAAA |
| *1770_EC_*qPCR_Rev | Reverse primer qRT-PCR | CCGTCTCCAGAATAGTCCGC |
| *0293_EC_*qPCR_Fwd | Forward primer qRT-PCR | TACCTCCCCTGTACGGTCC |
| *0293_EC_*qPCR_Rev | Reverse primer qRT-PCR | CGATACATTAGCGCTGCAGAG |
| *matB_EC_*qPCR_Fwd | Forward primer qRT-PCR | ATCGGTTACTGCGGTGGTAC |
| *matB_EC_*qPCR_Rev | Reverse primer qRT-PCR | ATGCCAGCAATCGTACCACT |
| *ygiL_EC_*qPCR_Fwd | Forward primer qRT-PCR | GTTGCTTTTAACGCGCTTGC |
| *ygiL_EC_*qPCR _Rev | Reverse primer qRT-PCR | GGAGCAATAGAACAAGGCGC |
| *afaA_EC_*qPCR_Fwd | Forward primer qRT-PCR | TCTGACTGACCCGCTGAAAC |
| *afaA_EC_*qPCR _Rev | Reverse primer qRT-PCR | TGCGCTGAGGGATTATCTGG |
| *4610_EC_*qPCR_Fwd | Forward primer qRT-PCR | TGTTGATTACCGGTAGCCTCG |
| *4610_EC_*qPCR _Rev | Reverse primer qRT-PCR | TCCTGCATCACTAACGGTAGC |
