# Supplementary material for: Yad fimbriae are triggered by host cues and enhance extraintestinal pathogenic Escherichia coli tissue colonisation during bloodstream infection
Source: PLoS Pathog. 2026 Jun 1;22(6):e1014299. doi: 10.1371/journal.ppat.1014299 (PMC13245861; doi:10.1371/journal.ppat.1014299)
Supplement: S4 Table — (DOCX) [file ppat.1014299.s013.docx]

**S4 Table** – Plasmids used in this study.

| **Plasmid** | **Description** | **Source** |
| --- | --- | --- |
| pMK1*lux* | pBR322 ori with the *luxCDABE* operon and MCS; Amp^R^ | Karavolos *et al.* 2008 *BMC Genomics* 6:458 |
| pMK1*lux*-P*_yad_* | pMK1*lux* with the CFT073 *yadN* promoter cloned *Eco*RI/*Bam*HI; Amp^R^ | This study |
| pSU2718 | pACYC184-derived cloning plasmid, Cm^R^ | Martinez *et al.* 1988 *Gene* 68:159-162 |
| pHNS-cm | pSU2718 with the *hns* coding sequence from EC958 cloned at *Hind*III | Tan *et al.* 2016 *PLoS ONE* 11(9):e0162391 |
| pSU-*yad* | pSU2718 with the *yad* operon coding sequence from EC958 cloned at *Hind*III | This study |
| pKD46 | Lambda Red recombinase expressing plasmid; temperature sensitive; Amp^R^ | Datsenko and Wanner, 2000 *PNAS* 97:6640-5 |
| pKD3 | Template plasmid for Lambda Red mutagenesis; Cm^R^ | Datsenko and Wanner, 2000 *PNAS* 97:6640-5 |
| pKD4 | Template plasmid for Lambda Red mutagenesis; Kan^R^ | Datsenko and Wanner, 2000 *PNAS* 97:6640-5 |
| pCP20 | FLP recombinase expressing plasmid; temperature sensitive; Amp^R^ | Datsenko and Wanner, 2000 *PNAS* 97:6640-5 |
| pCP20-Gent | FLP recombinase expressing plasmid; temperature sensitive; Gent^R^ | Cheraponov *et al.* 1995 *Gene* 158(1):9-14 |
